# Vitamin D deficiency and C-reactive protein: a bidirectional Mendelian randomization study

**DOI:** 10.1093/ije/dyac087

**Published:** 2022-05-17

**Authors:** Ang Zhou, Elina Hyppönen

**Affiliations:** Australian Center for Precision Health, University of South Australia Cancer Research Institute, Adelaide, Australia; South Australian Health and Medical Research Institute, Adelaide, Australia; Australian Center for Precision Health, University of South Australia Cancer Research Institute, Adelaide, Australia; South Australian Health and Medical Research Institute, Adelaide, Australia; Population, Policy and Practice, UCL Institute of Child Health, London, UK

**Keywords:** Non-linear Mendelian randomization, vitamin D, serum 25-hydroxyvitamin D concentration, C-reactive protein, chronic inflammation

## Abstract

**Background:**

Low vitamin D status is often associated with systemic low-grade inflammation as reflected by elevated C-reactive protein (CRP) levels. We investigated the causality and direction of the association between vitamin D status and CRP using linear and non-linear Mendelian randomization (MR) analyses.

**Methods:**

MR analyses were conducted using data from 294 970 unrelated participants of White-British ancestry from the UK Biobank. Serum 25-hydroxyvitamin D [25(OH)D] and CRP concentrations were instrumented using 35 and 46 genome-wide significant variants, respectively.

**Results:**

In non-linear MR analysis, genetically predicted serum 25(OH)D had an L-shaped association with serum CRP, where CRP levels decreased sharply with increasing 25(OH)D concentration for participants within the deficiency range (<25 nmol/L) and levelled off at ∼50 nmol/L of 25(OH)D (*P_non-linear_* = 1.49E-4). Analyses using several pleiotropy-robust methods provided consistent results in stratified MR analyses, confirming the inverse association between 25(OH)D and CRP in the deficiency range (*P* = 1.10E-05) but not with higher concentrations. Neither linear or non-linear MR analysis supported a causal effect of serum CRP level on 25(OH)D concentration (*P_linear_* = 0.32 and *P_non-linear_* = 0.76).

**Conclusion:**

The observed association between 25(OH)D and CRP is likely to be caused by vitamin D deficiency. Correction of low vitamin D status may reduce chronic inflammation.

Key Messages

- Our bidirectional Mendelian randomization (MR) analysis confirmed that the association between serum 25-hydroxyvitamin D [25(OH)D] and C-reactive protein (CRP) is likely to be driven by an effect of 25(OH)D on CRP rather than vice versa.
- Non-linear MR analyses showed that the effect of 25(OH)D on CRP is restricted to the vitamin D deficiency range, where higher genetically predicted 25(OH)D was associated with lower CRP concentrations.
- There was no evidence for an effect of genetically predicted serum CRP concentration on 25(OH)D in linear or non-linear MR analyses.
- Given that the serum CRP level is a widely used biomarker for chronic inflammation, these results suggest that improving vitamin D status may reduce chronic inflammation, but only for people with vitamin D deficiency.

## Introduction

Systemic low-grade inflammation, characterized by prolonged release of inflammatory mediators and activation of harmful signal-transduction pathways, is associated with various complex somatic and neuropsychiatric diseases and disorders.^1^^,^^2^ It is considered that nutritional factors can influence many aspects of inflammation.^3^ Vitamin D is a pro-hormone and an essential micronutrient, and although its classical roles are related to the regulation of calcium homeostasis, various types of immune cells express both the vitamin D receptor and metabolizing enzymes,^4^ suggesting that hormonal vitamin D could also play a role in modulating inflammatory responses.^5^ This is supported by an inverse association between serum 25-hydroxyvitamin D [25(OH)D] concentrations and C-reactive protein (CRP), which is frequently reported in observational studies.^6^^,^^7^ Serum 25(OH)D is the best indicator for vitamin D status whereas CRP is one of the most widely used inflammatory biomarkers in clinical practice. However, there is an ongoing debate about the causal nature of the association between 25(OH)D and CRP, and the observational association has not been supported by randomized trials.^8^ Indeed, it has been suggested that the association between serum 25(OH)D and CRP simply reflects reverse causality or confounding, where a low 25(OH)D concentration is either a consequence of chronic inflammation or results from behaviours such as less time outdoors in people who are unwell.^8^

Sitting at the interface between observational studies and randomized–controlled trials (RCTs), Mendelian randomization (MR) has been increasingly used to strengthen causal evidence in observational studies.^9^ It uses genetic variants associated with the exposure of interest to approximate the exposure and, conditional on the key method assumptions being met, MR has the benefit of reducing bias due to confounding and reverse causation.^9^ The association of 25(OH)D and CRP has previously been investigated using the MR approach, with no evidence to support a causal effect.^10^^,^^11^ However, all previous studies have only used the standard linear MR method, which cannot rule out the possibility of a threshold effect restricted to vitamin D deficiency.^12^ Indeed, it is logical to expect that improving vitamin D status would be relevant only in the presence of vitamin D deficiency, whereas any further additions may be redundant and, in the high extreme of supplementation, might become toxic.^13^ These types of non-linear dose–response relationships can be tested by the non-linear MR approach, which allows us to interrogate the shape of the association.^14^ This method has been recently used to provide evidence for the adverse effect of vitamin D deficiency on cardiovascular disease (CVD) risk and mortality, which is not visible using the standard linear MR approach.^15^^,^^16^ In this study we set out to examine evidence for the direction and causality of the association between serum 25(OH)D and CRP, also allowing for possible threshold effects. We performed these analyses using data from 294 970 participants in UK Biobank, representing the largest cohort to date with measured serum 25(OH)D concentrations.

## Methods

### Study participants

UK Biobank is a large prospective cohort study with >500 000 participants aged 37–73 years recruited from 22 assessment centres across the UK between 13 March 2006 and 1 October 2009 with a goal to improve the prevention, diagnosis and treatment of diseases of middle and old age.^17^ Participants filled in questionnaires to provide broad information on health and lifestyles at baseline survey and provided blood samples for biomarker and genetic assays. We restricted the analyses to unrelated individuals who were identified as White-British ancestry based on self-report and genetic profiling^18^ and excluded participants with mismatched information between self-reported and genetic sex. Final genetic analyses were conducted among individuals with complete information on serum 25(OH)D and CRP concentrations and relevant covariates (*N* = 294 970) (Supplementary Figure S1, available as Supplementary data at *IJE* online). The present study was conducted under UK Biobank application number 20175. The UK Biobank study was approved by the National Information Governance Board for Health and Social Care and North West Multicentre Research Ethics Committee (11/NW/0382). All participants provided informed consent to participate.

### Serum 25(OH)D and CRP concentrations

Blood samples of participants were collected at the time of recruitment. Serum 25(OH)D concentration (nmol/L) was measured using the LIAISON XL 25(OH)D assay (DiaSorin, Stillwater, USA) and serum CRP concentration (mg/L) was measured using high-sensitivity immunoturbidimetric assay on a Beckman Coulter AU5800^19^^,^^20^ (see Supplementary methods, available as Supplementary data at *IJE* online, for details). Since the distribution of serum CRP concentration is highly skewed, we natural-log-transformed serum CRP concentrations to facilitate analyses.

### Genetic instrument for serum 25(OH)D concentration

We constructed a weighted genetic score (vitaminD-GS) consisting of 35 single-nucleotide polymorphisms (SNPs) to instrument serum 25(OH)D concentration (Supplementary Table S1, available as Supplementary data at *IJE* online). All 35 SNPs are common genome-wide significant variants (minor allele frequency > 5%), discovered in a recent genome-wide association analysis (GWAS) for serum 25(OH)D concentration in UK Biobank^21^ and were replicated with a consistent direction and a *P*-value < 0.05 in the earlier GWAS by the SUNLIGHT consortium^22^ (Supplementary Figure S2, available as Supplementary data at *IJE* online). Benefits of replication in the SUNLIGHT consortium are 2-fold. It ensures the robustness of the GWAS signals and also allows us to take weights for vitaminD-GS from an independent sample, avoiding bias arising from using internal weights.^23^ VitaminD-GS was constructed by first computing the weighted average of the number of 25(OH)D-increasing alleles for an individual and then multiplying it by the number of available variants. The weight for each SNP was the effect estimate of the association of the SNP with serum 25(OH)D in the SUNLIGHT consortium.^22^ As a sensitivity analysis, we constructed an alternative instrument using a broader set of SNPs consisting of 122 autosomal variants (vitaminD-GS-122) (Supplementary methods, Supplementary Figure S2 and Supplementary Table S1, available as Supplementary data at *IJE* online). Further, to minimize any potential influence by horizontal pleiotropy (via variants with no clear biological links to vitamin D metabolism), in particular by metabolic traits (including lipids traits^24^), we included two additional instruments in the sensitivity analysis, which were constructed using only variants near/in genes related to vitamin D metabolism. A focused score^16^ consists of 21 variants from four loci, including *GC*, *DHCR7*, *CYP2R1* and *CYP24A1* (Supplementary methods, available as Supplementary data at *IJE* online). As *GC* and *CYP24A1* [which are involved in transport and clearance of 25(OH)D, respectively] are downstream of the production of 25(OH)D, their associations with high serum 25(OH)D concentrations may not accurately reflect concentrations of bioavailable 25(OH)D.^25^ As an attempt to better represent the level of ‘effective’ 25(OH)D, we repeated analyses using a synthesis score^26^^,^^27^ that only included variants from *DHCR7* and *CYP2R1*, which directly contribute to substrate availability and synthesis of 25(OH)D (Supplementary methods, available as Supplementary data at *IJE* online).

### Genetic instrument for serum CRP concentration

The genetic score for serum CRP concentration (CRP-gwasGS) was constructed using 46 genome-wide significant variants associated with serum CRP concentration, which were discovered in a recent GWAS meta-analysis with no overlap with UK Biobank.^28^ Information on these 46 variants can be found in Supplementary Table S2 (available as Supplementary data at *IJE* online). CRP-gwasGS was constructed in the same way as vitaminD-GS with the weight for each SNP being the effect estimate of the association of the SNP with CRP in the aforementioned meta-analysis.^28^ As a sensitivity analysis, we constructed an alternative instrument using four *cis*-acting variants^29^ (Supplementary Table S2, available as Supplementary data at *IJE* online).

### Statistical analysis

We performed linear and non-linear MR analyses to examine the genetic evidence for the bidirectional association between serum 25(OH)D and CRP concentrations. If a non-linear association was evident in the non-linear MR analysis, we also performed stratified MR analyses as a sensitivity analysis to gauge the robustness of the detected non-linearity. For the full analytical strategy, please see Supplementary Figure S3 (available as Supplementary data at *IJE* online). MR estimates of the linear, non-linear and stratified MR analyses were calculated using the genetic score-based approach (GS-based one-sample approach). For the linear and stratified analyses, where estimation via the SNP-based two-sample approach is also possible, we repeated the analyses applying five SNP-based two-sample methods as sensitivity analyses to gauge the robustness of the results to horizontal pleiotropy. Both GS-based one-sample and SNP-based two-sample approaches are described in detail below in the context of linear, stratified and non-linear MR analyses.

#### GS-based one-sample analysis for linear and stratified MR

In the GS-based one-sample analysis, the MR estimate is computed using the ratio-of-coefficients method,^30^ in which in the same sample (i.e. one-sample) GS–exposure and GS–outcome association estimates are computed and then taken as inputs to estimate the causal effect. For the linear MR analysis, the MR estimate is computed in the full sample, whereas in the stratified analysis, stratum-specific MR estimates are computed in the strata of residual exposure, which is defined as the exposure level minus the variation induced by the genetic score. Stratification is performed using residual exposure (rather than the raw exposure level) to avoid collider bias^31^^,^^32^ that could potentially induce spurious associations between GS and outcome, and bias stratum-specific MR estimates.

#### Non-linear MR analysis

The non-linear MR is also a GS-based one-sample approach and for our analysis we used the fractional polynomial method to capture the non-linearity of the exposure–outcome association.^14^ Briefly, the full UK Biobank sample was stratified into 100 strata using the residuals of exposure after regressing on the corresponding GS. Within each stratum, the localized average causal effect (LACE) was computed using the ratio-of-coefficients method, which is the ratio of the coefficient of the GS–outcome association estimate to that of the GS–exposure association estimate. Meta-regression of LACE against the stratum-specific mean exposure was then performed by fitting a range of fractional polynomial exposure–outcome models of Degrees 1 and 2, and the best-fitting model selected based on the likelihood ratio test. We report the fractional polynomial test for non-linearity in which the best-fitting fractional polynomial model of Degree 1 is compared against the linear model.^14^ Non-linear MR analyses assume that the GS–exposure association is constant over the entire distribution of exposure. To test this assumption, we examined the heterogeneity of GS–exposure associations across 100 strata using the Cochran’s Q test and trend test.^14^

#### SNP-based two-sample analysis for linear and stratified MR

We included five SNP-based two-sample methods in the linear and stratified MR analyses, including inverse-variance weighted (IVW), MR–Egger, weighted median, weighted mode and MR–Presso (Supplementary Figure S3, available as Supplementary data at *IJE* online). All these methods use individual SNPs (instead of an aggregate genetic score as in the GS-based approach) and take SNP–exposure and SNP–outcome association estimates as inputs. It is important to note that SNP–exposure and SNP–outcome association estimates need to be taken from independent samples (i.e. two-sample) otherwise bias can be introduced.^33^ The five SNP-based two-sample methods have largely independent assumptions on horizontal pleiotropy and a good agreement across these methods suggests a credible causal estimate (see Supplementary methods, available as Supplementary data at *IJE* online, for details). In the linear MR analysis, in which SNP-25(OH)D and SNP–CRP association estimates were all taken from UK Biobank, we applied the split-sample strategy to avoid bias due to overlapping samples.^33^ More specifically, the full UK Biobank sample is randomly split into two subsamples of equal size (Samples A and B); the MR estimate is calculated for each sample (MR_A_ and MR_B_) and then combined to obtain the overall estimate (MR_meta_) (Supplementary Figure S3, available as Supplementary data at *IJE* online). MR_A_ is computed using SNP–outcome association estimates from Sample A and SNP–exposure association estimates from Sample B, whereas SNP–outcome association estimates from Sample B and SNP–exposure association estimates from Sample A are used to compute MR_B_. MR_A_ and MR_B_ are then combined in a fixed-effects meta-analysis to compute MR_meta_. In the stratified analyses, the stratum-specific MR estimate is computed using SNP–outcome association estimates from the stratum under consideration with SNP–exposure association estimates taken from the full sample leaving out the stratum under consideration (Supplementary Figure S3, available as Supplementary data at *IJE* online).

Given that serum 25(OH)D and CRP concentrations are continuous, all GS/SNP–exposure/outcome association estimates required for the linear, stratified and non-linear analyses were computed by fitting linear regression models. All models were adjusted for age, sex, assessment centre, birth location, SNP array, top 40 genetic principal components and nuisance factors related to the measurement of serum 25(OH)D and/or CRP concentrations, including month in which blood sample was taken, fasting time before blood sample was taken and sample aliquots for measurement.^20^ Adjustment of birth location and 40 genetic components is recommended to account for latent population structure in UK Biobank.^34^ GS-based one-sample linear and stratified analyses were performed using STATA, version 17.0 (StataCorp LP, College Station, Texas, USA). SNP-based two-sample analyses and non-linear MR analysis were conducted in R (version 4.0.2) using the TwoSampleMR (version 0.5.6)^35^ and nlmr (version 2.0)^14^ package, respectively.

## Results

In total, 294 970 participants were included in the analysis (Supplementary Figure S1, available as Supplementary data at *IJE* online). The average 25(OH)D concentration was 50.0 nmol/L (range 10–340 nmol/L) and 11.7% (*n* = 34 403) of the participants had concentrations of <25 nmol/L (Table 1). There were notable variations in serum 25(OH)D and CRP concentrations with respect to distributions of demographics, lifestyle, general health and socio-economic factors (Table 1). Serum 25(OH)D and CRP concentrations are inversely related in a dose-dependent manner (*P *<* *1.0E-300) (Table 1).

**Table 1:** Serum 25(OH)D and CRP concentration by baseline characteristics in UK Biobank

		25(OH)D (nmol/L)	CRP (mg/L)
	*N* (%)	Mean (SD)	Gmean (GSD)
Age (years)			
<65	236 445 (80.16)	49.31 (21.01)	1.32 (2.90)
≥65	58 525 (19.84)	52.03 (20.57)	1.64 (2.78)
*P* ^a^		2.06E-188	<1.0E-300
Sex			
Male	138 911 (47.09)	49.92 (21.03)	1.34 (2.78)
Female	156 059 (52.91)	49.80 (20.89)	1.42 (2.98)
*P* ^a^		2.60E-03	<1.0E-300
BMI (kg/m^2^)			
<18.5	1443 (0.49)	50.72 (24.42)	0.56 (3.18)
(18.5–25)	96 053 (32.56)	53.01 (21.82)	0.86 (2.78)
(25–30)	125 559 (42.57)	50.61 (20.56)	1.41 (2.60)
≥30	71 037 (24.08)	44.32 (19.20)	2.57 (2.56)
Missing	878 (0.30)	42.53 (21.28)	2.23 (3.16)
*P* ^a^		<1.0E-300	9.36E-88
Smoking			
Non-smoker	160 897 (54.55)	50.07 (20.63)	1.27 (2.86)
Ex-smoker	103 600 35.12	50.81 (21.03)	1.47 (2.86)
Smoker^b^	7561 (2.56)	49.09 (21.57)	1.40 (2.94)
Cigars/pipe	1682 (0.57)	45.49 (21.08)	1.83 (2.71)
<1 to 15 cigs/day	12 056 (4.09)	45.36 (21.63)	1.73 (2.95)
>15 cigs/day	8158 (2.77)	41.55 (21.77)	2.35 (2.84)
Missing	1016 (0.34)	49.92 (21.76)	1.74 (2.90)
*P* ^a^		<1.0E-300	<1.0E-300
Alcohol intake			
Non-drinker	19 178 (6.50)	45.96 (20.95)	1.69 (3.06)
Special occasions or 1–3 times/month	63 647 (21.58)	47.17 (20.36)	1.62 (2.94)
1 or 2 times/week	78 199 (26.51)	50.44 (20.74)	1.38 (2.86)
3 or 4 times/week	71 296 (24.17)	51.41 (20.87)	1.23 (2.82)
Daily or almost daily	62 445 (21.17)	51.28 (21.51)	1.27 (2.82)
Missing	205 (0.07)	45.08 (21.27)	1.81 (2.98)
*P* ^a^		<1.0E-300	<1.0E-300
Physical activity			
Light	88 245 (29.92)	46.35 (20.20)	1.61 (2.92)
Moderate	142 803 (48.41)	50.64 (20.80)	1.28 (2.85)
Vigorous	57 373 (19.45)	54.02 (21.42)	1.24 (2.79)
Missing	6549 (2.22)	43.40 (21.30)	2.24 (3.09)
*P* ^a^		<1.0E-300	<1.0E-300
Education			
None	50 706 (17.19)	50.42 (21.42)	1.84 (2.82)
NVQ/CSE/A-levels	105 433 (35.74)	50.56 (21.18)	1.43 (2.87)
Degree/professional	136 357 (46.23)	49.08 (20.58)	1.21 (2.86)
Missing	2474 (0.84)	50.56 (20.96)	1.65 (2.88)
*P* ^a^		7.65E-87	<1.0E-300
TDI quartiles (min–max)			
Q1 (–6.26 to –3.76)	73 533 (24.93)	51.94 (20.72)	1.27 (2.81)
Q2 (–3.76 to –2.37)	73 780 (25.01)	51.53 (20.71)	1.33 (2.83)
Q3 (–2.37 to 0.030)	73 655 (24.97)	49.95 (20.77)	1.37 (2.88)
Q4 (0.030 to 10.82)	73 658 (24.97)	45.99 (21.08)	1.58 (3.0)
Missing	344 (0.12)	49.99 (20.46)	1.47 (3.14)
*P* ^a^		<1.0E-300	<1.0E-300
Self-rated health			
Excellent	49 237 (16.69)	53.06 (21.17)	0.98 (2.69)
Good	172 566 (58.50)	50.68 (20.71)	1.31 (2.78)
Fair	60 087 (20.37)	46.46 (20.63)	1.88 (2.91)
Poor	12 080 (4.10)	42.27 (21.18)	2.65 (3.17)
Missing	1000 (0.34)	43.70 (20.08)	1.91 (3.04)
*P* ^a^		<1.0E-300	<1.0E-300
Long-term illness			
No	194 251 (65.85)	50.76 (20.75)	1.23 (2.79)
Yes	94 129 (31.91)	48.11 (21.27)	1.74 (2.99)
missing	6590 (2.23)	47.96 (20.63)	1.58 (2.92)
*P* ^a^		<1.0E-300	<1.0E-300
25(OH)D (nmol/L)			
<25	34 403 (11.66)	–	1.64 (3.03)
25–50	121 812 (41.30)	–	1.44 (2.88)
50–75	103 400 (35.05)	–	1.30 (2.82)
>75	35 355 (11.99)	–	1.21 (2.91)
*P* ^a^		–	<1.0E-300
CRP quartiles (mg/L, min–max)			
Q1 (0.08–0.64)	72 663 (24.63)	51.91 (21.30)	–
Q2 (0.65–1.3)	74 111 (25.12)	50.97 (20.81)	–
Q3 (1.31–2.72)	74 359 (25.21)	49.33 (20.56)	–
Q4 (2.73–79.95)	73 837 (25.03)	47.24 (20.86)	–
*P* ^a^		<1.0E-300	–

CRP, C-reactive protein; 25(OH)D, 25-hydroxyvitamin D; BMI, body mass index; NVQ, National Vocational Qualification; CSE, Certificate of Secondary Education; A-levels, Advanced levels; SD, standard deviation; Gmean: geometric mean; GSD: geometric standard deviation; Q, quartiles; cig, cigarette; TDI, Townsend deprivation index.

a *P*-values have been adjusted for age, sex, assessment centre and nuisance factors that could affect serum 25(OH)D measurements, including month in which blood sample was taken, fasting time before blood sample was taken and sample aliquots for measurement.

b Current smokers without information on types of tobacco that they smoke.

### Instrument validation for vitaminD-GS

VitaminD-GS was robustly associated with serum 25(OH)D concentration in UK Biobank, explaining 2.8% of variation (F-statistic = 8646, *P *<* *1.0E-300) (Supplementary Figure S4, available as Supplementary data at *IJE* online). We examined the association of vitaminD-GS with serum 25(OH)D across 100 strata of residuals of serum 25(OH)D. We detected evidence for heterogeneity (*P_Cochran’s Q_* < 1.0E-300; *P_trend_* = 0.23), where vitaminD-GS–25(OH)D association in the 1st and 100th strata appeared to be outliers (Supplementary Figure S5, available as Supplementary data at *IJE* online). We found an association between the genetic instrument with serum low-density lipoprotein (LDL) and triglyceride concentrations (*P *<* *0.001 for both). In contrast there was no evidence that vitaminD-GS was associated with other potential confounders in UK Biobank, including body mass index (BMI), smoking, alcohol intake, physical activity, education and Townsend deprivation index (uncorrected *P* ≥ 0.051 for all) (Supplementary Table S3, available as Supplementary data at *IJE* online). We also examined vitaminD-GS–confounder associations across 100 strata of residuals of serum 25(OH)D and found no evidence of association after accounting for multiple testing (Supplementary Figure S6, available as Supplementary data at *IJE* online).

### Instrument validation for CRP-gwasGS

CRP-gwasGS explained 6.0% of variation in the serum log CRP concentration with a F-statistic of 18 687 (*P *<* *1.0E-300) (Supplementary Figure S7, available as Supplementary data at *IJE* online). There was evidence of heterogeneity in the CRP-gwasGS–CRP association across 100 strata of residuals of serum CRP, with the 1st and 100th strata appearing to be the outliers (*P_Cochran’s_*_Q_ = 2.66E-13, *P_trend_* = 0.28) (Supplementary Figure S8, available as Supplementary data at *IJE* online). CRP-gwasGS was not associated with the selected confounders, except for BMI (uncorrected *P *=* *1.066E-13) (Supplementary Table S3, available as Supplementary data at *IJE* online) and physical activity (uncorrected *P* = 0.004) (Supplementary Table S3, available as Supplementary data at *IJE* online). There was no evidence that CRP-gwasGS was associated with selected confounders across 100 strata of residuals of serum CRP concentrations after accounting for multiple testing (Supplementary Figure S9, available as Supplementary data at *IJE* online).

### MR of 25(OH)D on CRP

**Figure 1:**
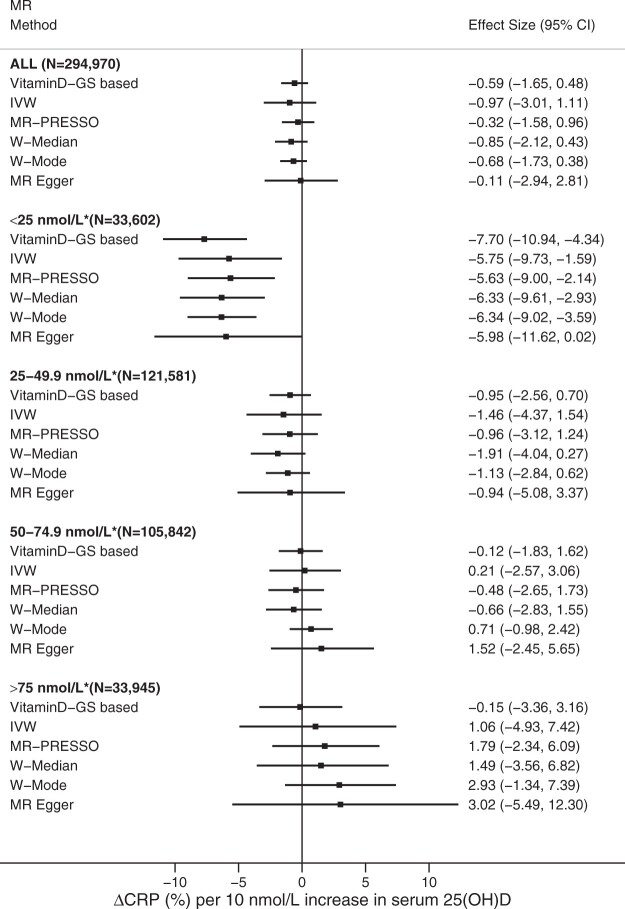
Linear and stratified MR analyses of serum 25(OH)D with CRP concentration. Adjustment includes age, sex, assessment centre, birth location, SNP array, top 40 genetic principal components and nuisance factors that could affect serum 25(OH)D and/or CRP measurements, including month in which blood sample was taken, fasting time before blood sample was taken and sample aliquots for measurement. CRP, C-reactive protein; 25(OH)D, 25-hydroxyvitamin D; IVW, inverse-variance weighted MR; W-Median, weighted median MR; W-Mode, weighted mode MR. *Residuals of serum 25(OH)D concentrations.

**Figure 2:**
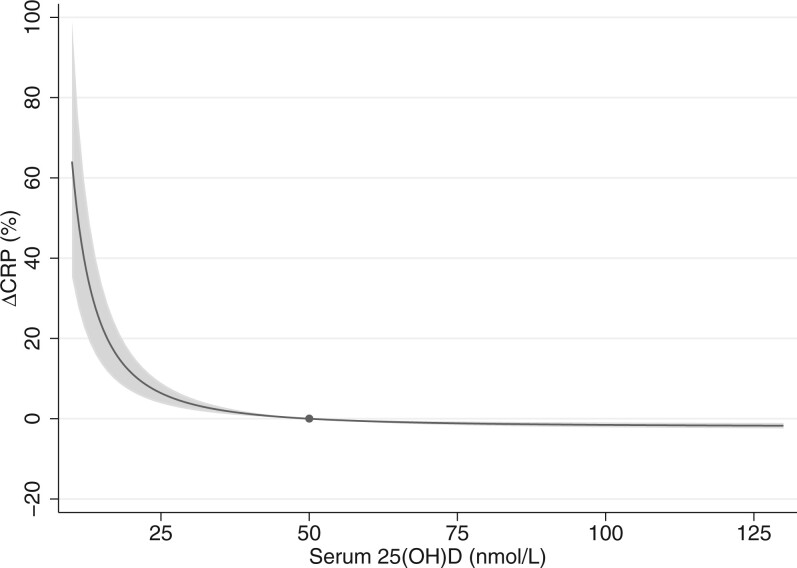
Non-linear MR analysis of serum 25(OH)D with CRP concentration. The dot represents the reference point of serum 25(OH)D of 50 nmol/L. The shaded areas represent the 95% confidence intervals. Adjustment includes age, sex, assessment centre, birth location, SNP array, top 40 genetic principal components and nuisance factors that could affect serum 25(OH)D and/or CRP measurement, including month in which blood sample was taken, fasting time before blood sample was taken and sample aliquots for measurement. CRP, C-reactive protein; 25(OH)D, 25-hydroxyvitamin D.

Linear MR analysis did not support a causal association of serum 25(OH)D with CRP concentration [–0.59% (95% CI, –1.65, 0.48) per 10-nmol/L increase in 25(OH)D, *P *=* *0.28) (Figure 1). The non-linear MR analysis suggested an ‘L-shaped’ association in which the serum CRP level peaked at the lowest 25(OH)D concentration, dropping steeply with increasing 25(OH)D concentration and levelling off at ∼50 nmol/L (*P_non-linear_* = 1.49E-4) (Figure 2). Compared with those with 50 nmol/L, individuals with serum 25(OH)D concentration at 25 nmol/L have a 6.4% (95% CI, 3.85–8.98) higher serum CRP concentration.

We conducted several sensitivity analyses to gauge the robustness of the detected non-linear association. First, we performed a stratified MR analysis using four categories of residual 25(OH)D concentration; <25.0, 25–49.9, 50.0–74.9 and ≥75.0 nmol/L. Consistently with the L-shaped association seen in the non-linear analysis, we only observed a genetic association of serum 25(OH)D with CRP concentration among individuals with residual 25(OH)D < 25 nmol/L, where each 10-nmol/L higher serum 25(OH)D concentration was associated with 7.70% lower CRP (95% CI, –10.9, –4.34, *P *=* *1.1E-05) (Figure 1). There was a good agreement in MR estimates across all five SNP-based two-sample methods within the <25-nmol/L strata (Figure 1). Further, as heterogeneity of the vitaminD-GS–25(OH)D association was detected (Supplementary Figure S5, available as Supplementary data at *IJE* online), we reanalysed the data excluding local causal estimates from the outlying strata of residuals of serum 25(OH)D (i.e. the 1st and 100th strata, after exclusion *P_Cochran’s Q_* = 0.30; *P_trend_* = 0.28), and this did not affect our findings (Supplementary Figure S10, available as Supplementary data at *IJE* online). Given the association between the vitamin-GS and blood lipids, we reanalysed the association adjusting for LDL and triglycerides, and restricting the instrument to variants that were not associated with any metabolic traits (a non-metabolic score) (Supplementary methods, available as Supplementary data at *IJE* online). Both approaches replicated the L-shaped association between 25(OH)D and CRP (Supplementary Figure S11, available as Supplementary data at *IJE* online) (*P_non-linear_* = 1.4E-03 for LDL adjustment, *P_non-linear_* = 0.057 for triglyceride adjustment and *P_non-linear_* = 1.6E-03 for the non-metabolic score). We also conducted reanalyses of non-linear MR using alternative instruments including vitaminD-GS-122 (*P_non-linear_* = 7.7E-07) (Supplementary Figure S12, available as Supplementary data at *IJE* online), focused score (*P_non-linear_* = 6.8E-10) (Supplementary Figure S13, available as Supplementary data at *IJE* online) and synthesis score (*P_non-linear_* = 3.3E-07) (Supplementary Figure S14, available as Supplementary data at *IJE* online) and found similar results.

### MR of CRP on 25(OH)D

Neither linear nor non-linear MR analyses provided any support for a causal effect of serum CRP on 25(OH)D concentration (*P_linear_* = 0.32 and *P_non-linear_* = 0.76) (Supplementary Table S4, available as Supplementary data at *IJE* online).

We performed several sensitivity analyses to gauge whether the null association in the linear and non-linear MR analyses could be related to the study design. First, as there is some evidence that CRP-gwasGS is associated with BMI and physical activity in UK Biobank (Supplementary Table S3, available as Supplementary data at *IJE* online), we re-performed linear and non-linear analyses adjusting for BMI, physical activity or both BMI and physical activity, and found similar results (Supplementary Table S4, available as Supplementary data at *IJE* online). Further, for the non-linear analysis, exclusion of local causal estimates from the outlying strata of residuals of serum CRP concentrations (i.e. the 1st and 100th strata, after exclusion *P*_Cochran’s Q_ = 0.51; *P*_trend_ = 0.28) did not affect our findings (*P_non-linear_* = 0.79) (Supplementary Table S4, available as Supplementary data at *IJE* online). Reanalyses using instrument with four *cis*-acting variants also provided similar results (Supplementary Table S4, available as Supplementary data at *IJE* online).

## Discussion

In this large-scale genetic analysis, we observed evidence for a causal effect of vitamin D status on CRP with no support for CRP as a determinant of 25(OH)D concentrations. The association between 25(OH)D and CRP was largely restricted to the deficiency range, where only individuals with low serum 25(OH)D concentrations have elevated serum CRP. The shape of the observed association supports the previously proposed threshold effect,^12^ suggesting that correction of vitamin D deficiency in the affected individuals is likely to reduce systemic low-grade inflammation and potentially mitigate the risk or severity of chronic illnesses with inflammatory components.

The earlier RCTs or MR studies have failed to provide evidence for an effect of vitamin D on CRP,^8^^,^^10^^,^^11^ which seemingly contradicts our finding. However, if the causal effect of vitamin D is truly L-shaped and restricted to concentrations within the deficiency range as seen in our study, it would have been overlooked both by the existing supplementation trials and linear MR studies. Severe deficiency is relatively rare^36^ and as it is unethical to subject participants to undue harm, supplementation trials often can only include individuals who are already vitamin D replete,^12^ rendering the health effect of supplementation in the deficiency range largely unassessed. Previous linear MR studies^10^^,^^11^ would have had limited statistical power to capture the threshold effect restricted to the deficiency range, which is indeed what was reflected in our linear MR analysis. We also found no evidence for an effect by genetically instrumented CRP on serum 25(OH)D concentration. This provides evidence against the notion that both molecules serve as acute phase reactants and that their association arises merely from confounding by inflammation.^37^ Indeed, if inflammation truly drives low serum 25(OH)D concentrations, we would have expected genetically instrumented CRP values to be associated with serum 25(OH)D concentration. Overall, our bidirectional MR analyses suggest that rather than vitamin D acting as a bystander [where 25(OH)D-CRP association arises merely from confounding by inflammation^37^], increasing 25(OH)D concentrations to alleviate a state of severe deficiency may help to mitigate the severity of inflammation. This said, it is important to note that these findings provide no support for a need to use high-dose vitamin D supplementation, as the observed benefits appeared to become largely saturated by the time 25(OH)D concentrations reach 50 nmol/L. It should also be noted that a higher vitamin D status may benefit some subpopulations or with respect to other disease outcomes,^38^ which is an area warranting further investigation.

Vitamin D is a pro-hormone. Its anti-inflammatory property captured by our analysis could be mediated through its hormonal effect on vitamin D receptor-expressing immune cells, such as monocytes, B cells, T cells and antigen-presenting cells.^4^ Indeed, cell experiments have shown that active vitamin D can inhibit the production of pro-inflammatory cytokines, including TNF-α, IL-1β, IL-6, IL-8 and IL-12, and promote the production of IL-10, an anti-inflammatory cytokine.^4^^,^^5^ Further, the anti-inflammatory effect also raises the possibility that having adequate vitamin D concentrations may mitigate complications arising from obesity and reduce the risk or severity of chronic illnesses with an inflammatory component, such as CVDs, diabetes, autoimmune diseases and neurodegenerative conditions, among others.^1^ If the related effects are indeed true, given the high prevalence of serum 25(OH)D levels of <50 nmol/L across the world (≤40% in some European countries),^36^^,^^39–42^ population-wide correction of low vitamin D status (e.g. by food fortification) could potentially be a cost-effective measure to reduce the burden of chronic disease. In fact, in linear MR analyses higher 25(OH)D concentrations have been associated with a lower risk of type 2 diabetes^43^ and multiple sclerosis (a chronic inflammatory disease of the central nervous system),^44^ with recent non-linear MR analyses providing evidence that correction of vitamin D deficiency can decrease the risk for CVDs^15^ and all-cause mortality.^16^

To the best of our knowledge, this is the first non-linear MR study to explore the bidirectional association between serum 25(OH)D and CRP concentrations. We used several strategies to ensure that our MR analyses are not affected by horizontal pleiotropy, where variants may influence the outcome through pathways other than through the exposure of interest.^9^ First, we restricted our vitaminD-GS to 35 variants with robust replicated evidence for an association with serum 25(OH)D concentrations. Second, we confirmed that the L-shaped association of serum 25(OH)D and CRP was consistent between the non-linear and stratified MR analyses, with the inverse association of serum 25(OH)D and CRP in the deficiency range consistently observed across pleiotropy-robust methods with largely independent assumptions on the pattern of pleiotropy. Third, the L-shaped association of serum 25(OH)D and CRP was confirmed to be robust using a spectrum of alternative instruments, including when using a broader set of variants, when excluding variants related to lipids and other metabolic traits and when restricting the set of variants to those directly related to vitamin D metabolism. Despite these strengths, our study also has some limitations. Although CRP is a widely used inflammatory biomarker, it certainly cannot capture the full complexity of the immune system and hence investigation of more specific biomarkers (such as TNF-α and IL-6) is required to provide a more detailed understanding on the anti-inflammatory effects of hormonal vitamin D. We restricted our analysis to participants of White-British descent to minimize bias due to population stratification; however, this may limit the transferability of our findings to other ethnic groups. As with all MR studies, genetic instruments approximate the average effects over the life course and the true biological association between serum 25(OH)D and CRP may be more complex than that indexed in our study. With only a 5% response rate at the recruitment, UK Biobank is not representative of the general public in the UK^45^ despite its large sample size. It is uncertain to what extent this selection could affect the non-linear MR analysis. However, given that risk factor–disease associations show close agreement between UK Biobank and nationally representative studies^46^ and that an earlier publication from UK Biobank using the same non-linear MR approach has replicated the expected J-shaped association between BMI and mortality,^47^ this lack of representativeness may not be affecting our findings.

## Conclusions

In conclusion, using a large population-based cohort, we provide genetic evidence for an L-shaped association of serum 25(OH)D with CRP, suggesting that the benefit of increasing 25(OH)D is restricted to individuals with low vitamin D status. Our finding suggests that improving vitamin D status in the deficiency range could reduce systemic low-grade inflammation and potentially mitigate the risk or severity of chronic illnesses with an inflammatory component.

## Ethics approval

The present study was conducted under UK Biobank application number 20175. The UK Biobank study was approved by the National Information Governance Board for Health and Social Care and North West Multicentre Research Ethics Committee (11/NW/0382).

## Supplementary Material

dyac087_Supplementary_Data

## Data Availability

Data are available from UK Biobank for researchers who meet the criteria and gain approvals to access the research database from the UK Biobank access management committee at the University of Oxford.
