# Supplementary material for: Vitamin D deficiency and C-reactive protein: a bidirectional Mendelian randomization study
Source: Int J Epidemiol. 2022 May 17;52(1):260–71. doi: 10.1093/ije/dyac087 (PMC9908047; doi:10.1093/ije/dyac087)
Supplement: dyac087_Supplementary_Data [file dyac087_supplementary_data.docx]

**Supplementary Material**

**Supplementary Methods**

**Measurement of serum 25(OH)D and CRP concentrations**

Serum 25(OH)D concentration (nmol/L) was measured using the LIAISON XL 25(OH)D assay (DiaSorin, Stillwater, USA), and serum CRP concentration (mg/L) was measured using high-sensitivity immunoturbidimetric assay on a Beckman Coulter AU5800 ^1^. The UK Biobank followed rigorous quality control protocols, including assessment of precision, accuracy, bias, linearity, reportable range, carryover, and multi-instrument comparison ^2^. Each assay was also registered with an external quality assurance scheme, and assay performance was externally verified via the results returned from participation in these scheme ^2^. The results of the external quality assurance were assessed to be Good or Acceptable across all submissions of 25(OH)D and CRP measurements ^1^. Participants with 25(OH)D concentrations below or above the validated range for the assay (10–375 nmol/L) were excluded. The average within-laboratory coefficient of variation (CV) (and standard deviation) for 25(OH)D concentrations ranged from 5.04 (4.73) to 6.14 (2.21) ^1^. For CRP concentrations, CV ranged from 1.69 (0.14) to 2.31 (0.02), with participants with values outside the range of 0.08–80 mg/L being excluded ^1^.

**Alternative genetic instruments for serum 25(OH)D concentration**

As a sensitivity analysis, we constructed 4 alternative genetic instruments for serum 25-hydroxyvitamin D (25(OH)D) concentrations, including vitaminD-GS-122, non-metabolic score, focused score and synthesis score. VitaminD-GS-122 was constructed using a broader set of variants consisting of 122 autosomal single nucleotide polymorphisms (SNPs, Supplementary Figure S2). Information for the 122 SNPs can be found in Supplementary Table S2). As 122 SNPs were discovered in the UK Biobank ^3^, to mitigate any bias induced by using the internal weights, we used the 10-fold cross-validation approach ^4^ to construct the vitaminD-GS-122, where the sample was divided randomly into 10 equal sub-samples, with weights for each sub-sample taken from the other 9 sub-samples. The non-metabolic score was constructed using the same set of variants as vitaminD-GS but with the exclusion of 11 variants associated with metabolic traits. The metabolic-traits-related variants were identified through the PhenoScanner V2 ^5^ (accessed on 16/02/2022), which is a curated database of publicly available results from large-scale genetic association studies. Among 35 variants that were used to constructed vitaminD-GS, 11 variants were associated with one or more metabolic traits at the GWAS threshold (*P*<5E-08) (Supplementary Table S5). The focused score was constructed using 21 variants from four loci related to vitamin D metabolism, including *GC*, *DHCR7*, *CYP2R1* and *CYP24A1* ^6^. It was weighted by the conditional associations of the genetic variants with 25(OH)D concentration in the UK Biobank (Supplementary Table S6). Information for all 21 SNPs can be found in Supplementary Table S6. The synthesis score was constructed by summing the number of serum-25(OH)D-increasing alleles of two variants from *DHCR7* (rs12785878) and *CYP2R1* (rs12794714) ^7,8^.

**SNP-based two-sample methods**

For the SNP-based two-sample analysis, we included five methods, including inverse variance weighted (IVW), MR-Egger, weighted median, weighted mode, and MR-Presso (Supplementary Figure S3). Each method is robust to different patterns of horizontal pleiotropy, and therefore a good agreement across all methods suggests strong evidence of causal association. IVW will return an unbiased causal estimate in the absence of directional pleiotropy ^9^. MR-Egger allows for directional pleiotropy (at the cost of statistical power), although it is still prone to a particular type of pleiotropic pattern when the instrument is associated with confounders of exposure and outcome ^10^. Weighted median uses the weighted median of the ratio estimates, and requires that set of variants accounting for 50% or more of the total weight is valid ^11^. Weighted mode will return an unbiased estimate if variants within the cluster that has the largest weighted number of variants are valid ^12^. MR-Presso performs an outlier test to detect and remove potentially pleiotropic outlier variants. If no outliers detected it will return the same estimate as IVW ^13^.


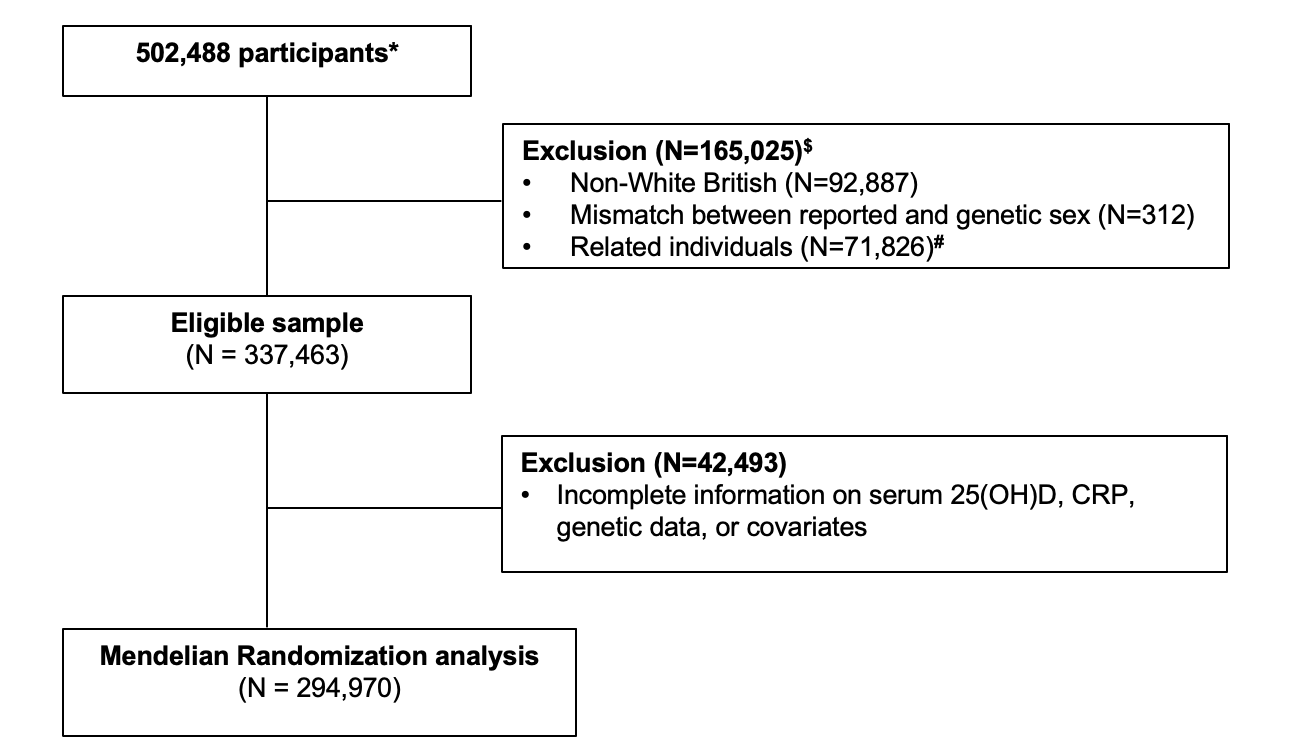


**Figure S1. Participant flow chart of the study.** *Excludes participants who withdrew consent after participating in the study. ^$^Exclusion was done in a sequential order. ^#^Patterns of relatedness were identified from genotyping data; In the current study we allowed for one member from each family

**
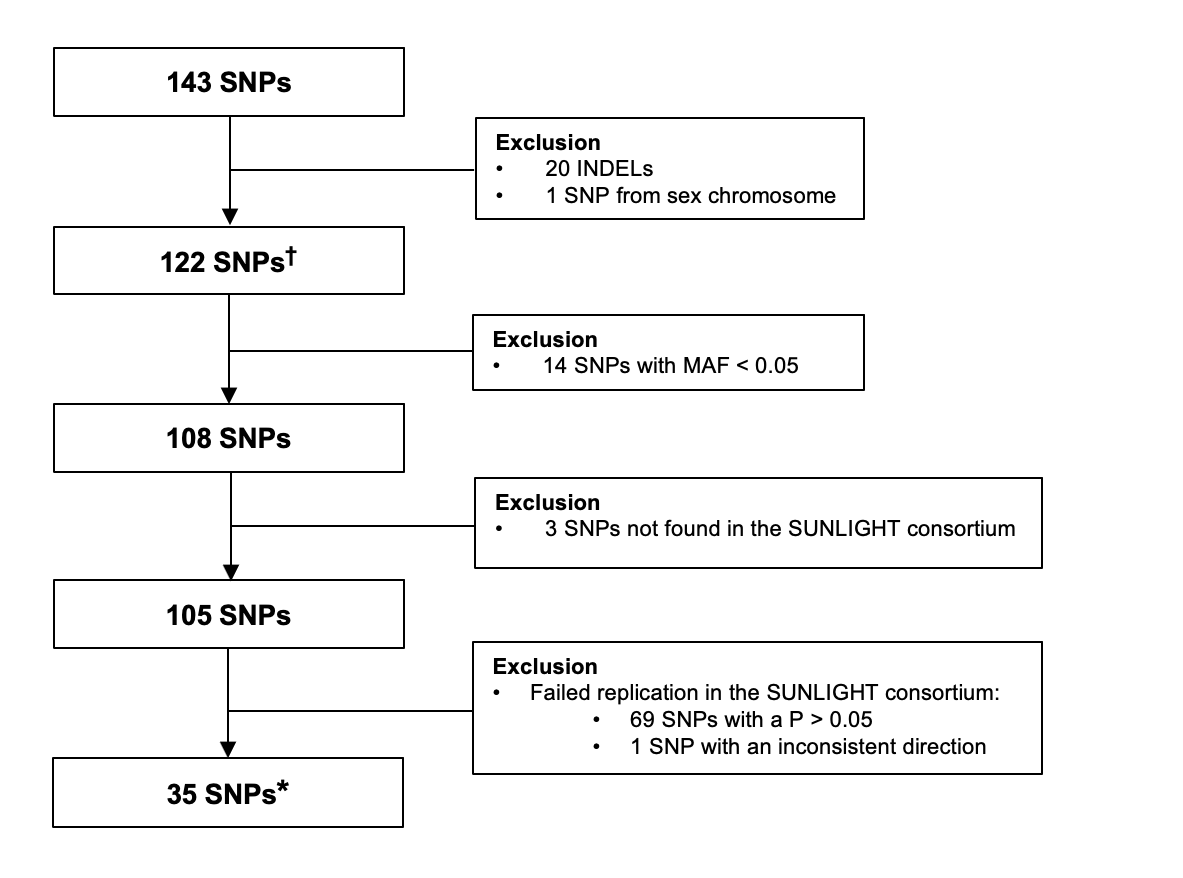
**

**Figure S2.** **Selection of variants for the genetic instrument for serum 25(OH)D.** *SNPs used for vitaminD-GS in the primary analysis; ^†^SNPs used for vitaminD-GS-122 in the sensitivity analysis. SNP: single nucleotide polymorphism; INDEL: insertion and deletion; MAF: minor allele frequency; 25(OH)D: 25-hydroxyvitamin D.


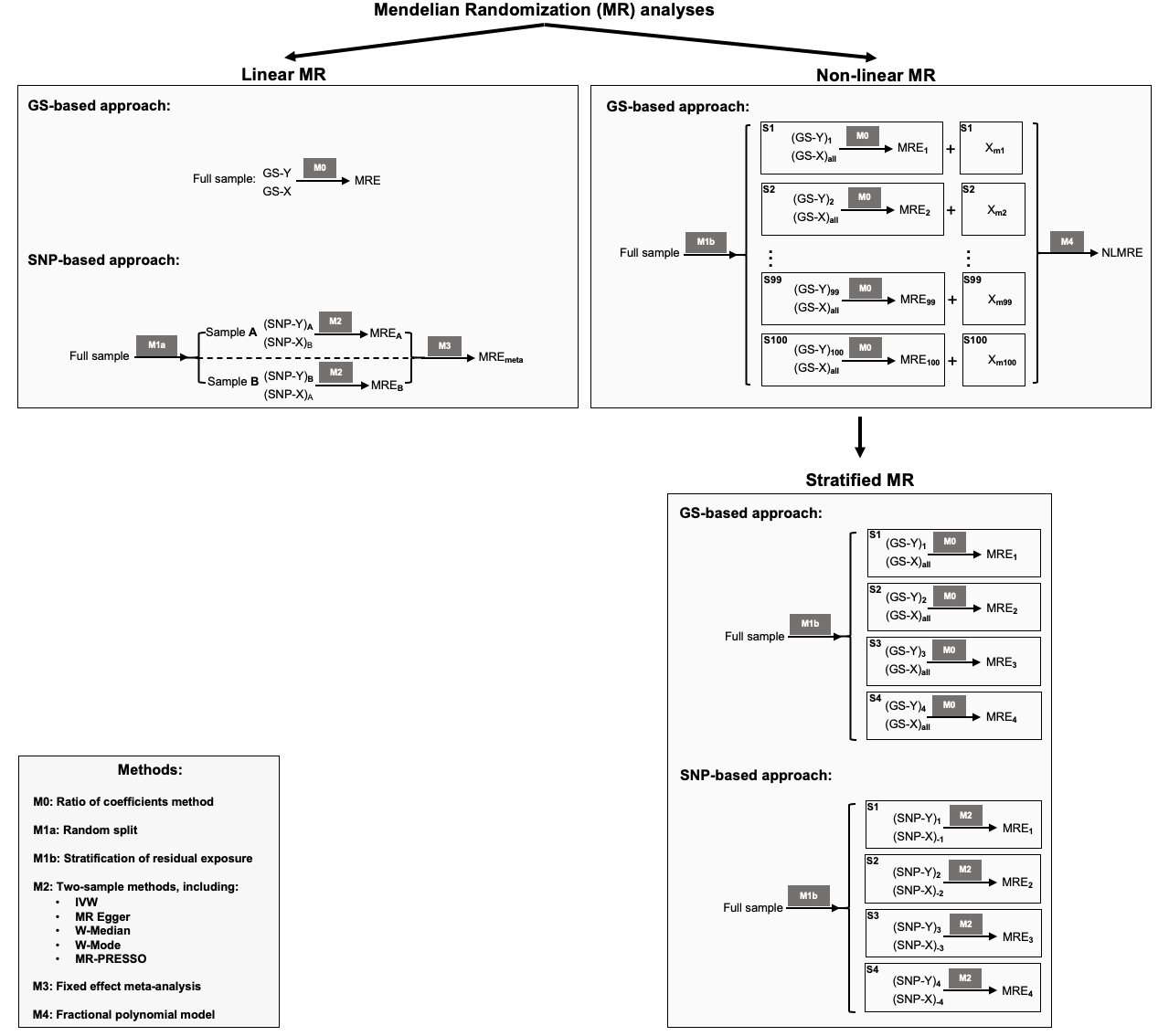


**Figure S3: Schematic representation of Mendelian randomization analyses performed in the current study.** GS: genetic score; SNP: single nucleotide polymorphism; S_n_: the n^th^ stratum; MRE: MR estimates; NLMRE: non-linear MR estimates; GS-X: beta of GS-exposure association; GS-Y: beta of GS-outcome association; SNP-X: beta of SNP-exposure association; SNP-Y: beta of SNP-outcome association; For linear MR, SNP-based approach: (SNP-X)_A/B_: SNP-X from sample A or B; (SNP-Y)_A/B_: SNP-Y from sample A or B; For stratified and non-linear MR, GS-based approach: (GS-X)_all_: GS-X from the full sample; (GS-Y)_n_: GS-Y from the n^th^ stratum; X_mn_: median of exposure in the n^th^ stratum; For stratified MR, SNP-based approach: (SNP-Y)_n_: SNP-Y from the nth stratum; (SNP-X)_-n_: SNP-X from the full sample excluding the n^th^ stratum.

**Figure S4.** **Distribution of vitamin D-GS and the association of sextiles of vitaminD-GS with serum 25(OH)D concentration in the UK Biobank, illustrating the validity of the genetic instrument.** The association was tested using the linear regression, with the model adjusted for age, sex, genotyping array, birth location, assessment center location, top 40 genetic principal components, and nuisance factors which could affect serum 25(OH)D measurements, including month in which blood sample was taken, fasting time before blood sample was taken, and sample aliquots for measurement. Error bars are 95% confidence intervals. Vitamin D-GS: genetic score for serum 25(OH)D concentration; 25(OH)D: 25-hydroxyvitamin D.

**Figure S5.** **Distribution of** $\boldsymbol{\beta}$**s for vitaminD-GS - 25(OH)D association across 100 strata of residuals of serum 25(OH)D**. Error bars are 95% confidence intervals. 25(OH)D: 25-hydroxyvitamin D.

**Figure S6.** **Association of vitaminD-GS with potential confounders across 100 strata of residuals of serum 25(OH)D.** Potential confounders include BMI, alcohol intake, smoking status, physical activity level, education attainment and Townsend deprivation index. *P_threshold_*=0.05/(6 confounders x 100 strata) = 8.3E-5. Dashed line represents -log_10_P at *P_threshold_*. 25(OH)D: 25-hydroxyvitamin D; BMI: Body mass index; TDI: Townsend deprivation index.

**Figure S7.** **Distribution of CRP-gwasGS and the association of sextiles of CRP-gwasGS with serum CRP concentration in the UK Biobank, illustrating the validity of the genetic instrument.** The association was tested using the linear regression, with the model adjusted for age, sex, genotyping array, birth location, assessment center location, top 40 genetic principal components, and nuisance factors which could affect serum CRP measurement, including month in which blood sample was taken, fasting time before blood sample was taken, and sample aliquots for measurement. Error bars are 95% confidence intervals. CRP: C-reactive protein: CRP-gwasGS: genetic score for serum CRP concentration using 46 genomewide variants; 25(OH)D: 25-hydroxyvitamin D.

**Figure S8.** **Distribution of** $\boldsymbol{\beta}$**s for CRP-gwasGS - logCRP association across 100 strata of residuals of serum logCRP concentrations**. Error bars are 95% confidence intervals. CRP: C-reactive protein.

**Figure S9.** **Association of CRP-gwasGS with potential confounders across 100 strata of residuals of serum logCRP concentrations.** Potential confounders include BMI, alcohol intake, smoking status, physical activity level, education attainment and Townsend deprivation index. *P_threshold_* = 0.05/(6 confounders x 100 strata) = 8.3E-5. Dashed line represents -log_10_P at *P_threshold_*. CRP: C-reactive protein; CRP-gwasGS: genetic score for serum CRP concentration using 46 genomewide variants; BMI: Body mass index; TDI: Townsend deprivation index.

**Figure S10.** **Non-linear MR analysis of serum 25(OH)D with CRP, after excluding local causal estimates from the first and 100^th^ stratum of residuals of serum 25(OH)D.** The dot represents the reference point of serum 25(OH)D of 50nmol/L. The shaded areas represent the 95% confidence intervals. The adjustment includes age, sex, assessment center, birth location, SNP array, top 40 genetic principal components, and nuisance factors which could affect serum 25(OH)D and/or CRP measurement, including month in which blood sample was taken, fasting time before blood sample was taken, and sample aliquots for measurement. CRP: C-reactive protein.

**A**

**B**

**C**

**Figure S11:** **Non-linear MR analysis of serum 25(OH)D with CRP concentration, (A) using vitaminD-GS + LDL adjustment (B) using vitaminD-GS + triglycerides adjustment (C) using the non-metabolic score.** The dot represents the reference point of serum 25(OH)D of 50nmol/L. The shaded areas represent the 95% confidence intervals. Adjustment includes age, sex, assessment center, birth location, SNP array, top 40 genetic principal components, and nuisance factors which could affect serum 25(OH)D and/or CRP measurement, including month in which blood sample was taken, fasting time before blood sample was taken, and sample aliquots for measurement. CRP: C-reactive protein; 25(OH)D: 25-hydroxyvitamin D; LDL: low density lipoprotein.

**Figure S12.** **Non-linear MR analysis of serum 25(OH)D with CRP, using vitaminD-GS-122.** The dot represents the reference point of serum 25(OH)D of 50nmol/L. The shaded areas represent the 95% confidence intervals. The adjustment includes age, sex, assessment center, birth location, SNP array, top 40 genetic principal components, and nuisance factors which could affect serum 25(OH)D and/or CRP measurement, including month in which blood sample was taken, fasting time before blood sample was taken, and sample aliquots for measurement. CRP: C-reactive protein; 25(OH)D: 25-hydroxyvitmain D.

**Figure S13:** **Non-linear MR analysis of serum 25(OH)D with CRP concentration, using the focused score.** The dot represents the reference point of serum 25(OH)D of 50nmol/L. The shaded areas represent the 95% confidence intervals. Adjustment includes age, sex, assessment center, birth location, SNP array, top 40 genetic principal components, and nuisance factors which could affect serum 25(OH)D and/or CRP measurement, including month in which blood sample was taken, fasting time before blood sample was taken, and sample aliquots for measurement. CRP: C-reactive protein; 25(OH)D: 25-hydroxyvitamin D.

**Figure S14:** **Non-linear MR analysis of serum 25(OH)D with CRP concentration, using the synthesis score.** The dot represents the reference point of serum 25(OH)D of 50nmol/L. The shaded areas represent the 95% confidence intervals. Adjustment includes age, sex, assessment center, birth location, SNP array, top 40 genetic principal components, and nuisance factors which could affect serum 25(OH)D and/or CRP measurement, including month in which blood sample was taken, fasting time before blood sample was taken, and sample aliquots for measurement. CRP: C-reactive protein; 25(OH)D: 25-hydroxyvitamin D

**Table S1. Genomewide significant vitamin D variants used for the genetic instruments for serum 25(OH)D concentrations.**

|  |  |  |  |  |  |  |  | **UK Biobank^a^** | | | **SUNLIGHT Consortium^b^** | | | **SNPs for** | **SNPs for** |
| --- | --- | --- | --- | --- | --- | --- | --- | --- | --- | --- | --- | --- | --- | --- | --- |
| **No** | **SNP** | **CHR** | **BP** | **Gene** | **A1** | **A2** | **A1F** | **Beta** | **SE** | ***P*** | **Beta** | **SE** | ***P*** | **vitaminD-GS** | **vitaminD-GS-122** |
| 1 | rs6671730^c^ | 1 | 2339139 | *PEX10* | G | A | 0.565714 | 0.0147881 | 0.00201077 | 1.92E-13 | 0.0061 | 0.0023 | 0.006652 | Yes | Yes |
| 2 | rs35408430 | 1 | 17560195 | *PADI1* | C | T | 0.657806 | 0.0214952 | 0.00209979 | 1.36E-24 | 0.0236985 | 0.00564768 | 0.00002715 | Yes | Yes |
| 3 | rs7522116 | 1 | 41835685 | *FOXO6* | C | T | 0.433767 | 0.0134641 | 0.00202533 | 2.97E-11 | 0.0116727 | 0.00540416 | 0.03077654 | Yes | Yes |
| 4 | rs7528419 | 1 | 109817192 | *CELSR2* | G | A | 0.224671 | 0.0197401 | 0.00238729 | 1.35E-16 | 0.0179046 | 0.00644566 | 0.0054732 | Yes | Yes |
| 5 | rs1933064 | 1 | 152301576 | *FLG-AS1* | A | G | 0.46961 | 0.015731 | 0.00203195 | 9.80E-15 | 0.0155068 | 0.00539368 | 0.00404027 | Yes | Yes |
| 6 | rs76798800 | 1 | 154994978 | *DCST2* | G | T | 0.733745 | 0.0121989 | 0.00225962 | 6.71E-08 | 0.0173898 | 0.00617041 | 0.00482841 | Yes | Yes |
| 7 | rs6672758 | 1 | 230303512 | *GALNT2* | T | C | 0.800872 | 0.0175857 | 0.00250898 | 2.40E-12 | 0.0156301 | 0.00666121 | 0.01895423 | Yes | Yes |
| 8 | rs727857 | 2 | 58981967 | *LINC01122* | G | A | 0.388511 | 0.0140184 | 0.00206152 | 1.05E-11 | 0.0109131 | 0.00550772 | 0.04754487 | Yes | Yes |
| 9 | rs1047891 | 2 | 211540507 | *CPS1* | C | A | 0.684179 | 0.0152142 | 0.00214041 | 1.18E-12 | 0.0126572 | 0.00572581 | 0.02706743 | Yes | Yes |
| 10 | rs2012736 | 2 | 234622379 | *UGT1A5, UGT1A6, UGT1A7, UGT1A8, UGT1A9, UGT1A10* | C | A | 0.919186 | 0.0483073 | 0.00366555 | 1.16E-39 | 0.0384413 | 0.01038238 | 0.00021344 | Yes | Yes |
| 11 | rs6782190 | 3 | 85639672 | *CADM2* | G | A | 0.352488 | 0.0172156 | 0.00208415 | 1.45E-16 | 0.0206718 | 0.00562451 | 0.00023756 | Yes | Yes |
| 12 | rs705117 | 4 | 72608115 | *GC* | C | T | 0.1477 | 0.0334179 | 0.00280601 | 1.06E-32 | 0.0269429 | 0.00744137 | 0.00029382 | Yes | Yes |
| 13 | rs1352846 | 4 | 72617775 | *GC* | A | G | 0.708567 | 0.193471 | 0.00219074 | 0 | 0.2221843 | 0.00589771 | 1.40E-310 | Yes | Yes |
| 14 | rs78151190 | 6 | 25619007 | *CARMIL1 (LRRC16A)* | A | C | 0.871284 | 0.0168754 | 0.00297406 | 1.39E-08 | 0.0187117 | 0.00829773 | 0.02413132 | Yes | Yes |
| 15 | rs75741381 | 7 | 100809458 | *VGF* | C | G | 0.852362 | 0.0166065 | 0.00282521 | 4.15E-09 | 0.0214474 | 0.00736197 | 0.00357669 | Yes | Yes |
| 16 | rs12056768 | 8 | 116988527 | *LINC00536* | T | G | 0.417091 | 0.0234029 | 0.00202418 | 6.44E-31 | 0.0176616 | 0.00545433 | 0.00120331 | Yes | Yes |
| 17 | rs77532868 | 10 | 88081438 | *GRID1* | T | C | 0.054042 | 0.0265692 | 0.00440069 | 1.57E-09 | 0.0280553 | 0.01353113 | 0.03813628 | Yes | Yes |
| 18 | rs12794714 | 11 | 14913575 | *CYP2R1* | G | A | 0.578197 | 0.0878964 | 0.00201629 | 0 | 0.0702488 | 0.00540376 | 1.22E-38 | Yes | Yes |
| 19 | rs61891388 | 11 | 66079818 | *RP11-867G23.13* | G | T | 0.455921 | 0.0125532 | 0.00200799 | 4.06E-10 | 0.0114254 | 0.00538961 | 0.03401532 | Yes | Yes |
| 20 | rs1660839 | 11 | 71094232 | *AP002387.1* | A | G | 0.248849 | 0.0292665 | 0.00230557 | 6.40E-37 | 0.014173 | 0.00623639 | 0.02304867 | Yes | Yes |
| 21 | rs12803256 | 11 | 71132868 | *AP002387.1* | G | A | 0.776732 | 0.104243 | 0.00239998 | 0 | 0.0839119 | 0.00602549 | 4.39E-44 | Yes | Yes |
| 22 | rs12798050^d^ | 11 | 71223256 | *S100A11P3* | T | C | 0.830503 | 0.109998 | 0.00264849 | 0 | 0.0348 | 0.0024 | 1.00E-47 | Yes | Yes |
| 23 | rs72997623 | 11 | 75488054 | *DGAT2* | A | C | 0.084662 | 0.0276158 | 0.00358139 | 1.25E-14 | 0.0200157 | 0.00937765 | 0.03280964 | Yes | Yes |
| 24 | rs1149605 | 11 | 76485216 | *RP11-21L23.4* | C | T | 0.170397 | 0.0220166 | 0.00266133 | 1.31E-16 | 0.0209786 | 0.0072426 | 0.00377288 | Yes | Yes |
| 25 | rs10859995 | 12 | 96375682 | *HAL* | T | C | 0.417366 | 0.0403465 | 0.0020206 | 1.05E-88 | 0.036551 | 0.00540543 | 1.36E-11 | Yes | Yes |
| 26 | rs8018720 | 14 | 39556185 | *SEC23A* | G | C | 0.176673 | 0.0378247 | 0.00260904 | 1.26E-47 | 0.040852 | 0.00705183 | 6.91E-09 | Yes | Yes |
| 27 | rs261291 | 15 | 58680178 | *ALDH1A2* | T | C | 0.644772 | 0.0273653 | 0.00208561 | 2.50E-39 | 0.0113468 | 0.0056366 | 0.04410853 | Yes | Yes |
| 28 | rs77924615 | 16 | 20392332 | *PDILT* | G | A | 0.806515 | 0.0166321 | 0.00255158 | 7.11E-11 | 0.0195535 | 0.00670691 | 0.00355194 | Yes | Yes |
| 29 | rs212100 | 19 | 48376995 | *SULT2A1* | T | C | 0.164001 | 0.0661522 | 0.00269018 | 1.61E-133 | 0.0193875 | 0.00719712 | 0.00706453 | Yes | Yes |
| 30 | rs10426 | 19 | 51517798 | *KLK10* | A | G | 0.213433 | 0.0256629 | 0.00243056 | 4.64E-26 | 0.0146449 | 0.0065379 | 0.02509092 | Yes | Yes |
| 31 | rs6123359 | 20 | 52714706 | *BCAS1* | G | A | 0.102225 | 0.0341831 | 0.00331429 | 6.10E-25 | 0.0373636 | 0.00940288 | 0.00007078 | Yes | Yes |
| 32 | rs17216707 | 20 | 52732362 | *CYP24A1* | T | C | 0.817316 | 0.0376264 | 0.00263713 | 3.47E-46 | 0.0646902 | 0.0066412 | 2.02E-22 | Yes | Yes |
| 33 | rs2585442 | 20 | 52737123 | *CYP24A1* | G | C | 0.240654 | 0.0356675 | 0.00237687 | 6.70E-51 | 0.0381477 | 0.00635676 | 1.96E-09 | Yes | Yes |
| 34 | rs2762943 | 20 | 52790786 | *CYP24A1* | G | T | 0.923071 | 0.0457231 | 0.00373798 | 2.10E-34 | 0.032534 | 0.01442103 | 0.02406994 | Yes | Yes |
| 35 | rs2074735 | 22 | 31535872 | *PLA2G3* | C | G | 0.064096 | 0.0278196 | 0.00407045 | 8.23E-12 | 0.0213678 | 0.01054517 | 0.04273241 | Yes | Yes |
| 36 | rs11591147 | 1 | 55505647 | *PCSK9* | T | G | 0.018041 | 0.0450903 | 0.00747745 | 1.64E-09 | - | - | - | No | Yes |
| 37 | rs2131925 | 1 | 63025942 | *DOCK7* | G | T | 0.356375 | 0.0229402 | 0.0020845 | 3.61E-28 | - | - | - | No | Yes |
| 38 | rs140371183 | 1 | 152098428 | *PUDPP2 (HDHD1P2)* | G | A | 0.032179 | 0.0870147 | 0.00565335 | 1.86E-53 | - | - | - | No | Yes |
| 39 | rs12123821 | 1 | 152179152 | *FLG-AS1, RP11-107M16.2* | T | C | 0.047527 | 0.0785529 | 0.00467652 | 2.55E-63 | - | - | - | No | Yes |
| 40 | rs61816761 | 1 | 152285861 | *FLG-AS1,FLG* | A | G | 0.015926 | 0.12315 | 0.00804767 | 7.35E-53 | - | - | - | No | Yes |
| 41 | rs10908419 | 1 | 154567699 | *ADAR* | G | A | 0.510067 | 0.012342 | 0.00199206 | 5.81E-10 | - | - | - | No | Yes |
| 42 | rs11264322 | 1 | 155087933 | *Y_RNA* | G | A | 0.570235 | 0.0093848 | 0.0020223 | 3.47E-06 | - | - | - | No | Yes |
| 43 | rs10908465 | 1 | 155389688 | *ASH1L* | T | C | 0.267332 | 0.0168816 | 0.00224924 | 6.12E-14 | - | - | - | No | Yes |
| 44 | rs867772 | 1 | 220972343 | *MARC1* | A | G | 0.315487 | 0.0146031 | 0.00215141 | 1.14E-11 | - | - | - | No | Yes |
| 45 | rs7604788 | 2 | 21190024 | *RP11-116D2.1* | T | C | 0.033433 | 0.0336737 | 0.0055518 | 1.32E-09 | - | - | - | No | Yes |
| 46 | rs541041 | 2 | 21294975 | *APOB* | G | A | 0.180761 | 0.0154585 | 0.00258635 | 2.27E-09 | - | - | - | No | Yes |
| 47 | rs1260326 | 2 | 27730940 | *GCKR* | C | T | 0.606565 | 0.0206128 | 0.00203644 | 4.41E-24 | - | - | - | No | Yes |
| 48 | rs11127186 | 2 | 28881407 | *AC074011.2* | C | T | 0.495795 | 0.0109023 | 0.00203379 | 8.30E-08 | - | - | - | No | Yes |
| 49 | rs2710651 | 2 | 63166379 | *EHBP1* | G | A | 0.471877 | 0.0114568 | 0.00199623 | 9.51E-09 | - | - | - | No | Yes |
| 50 | rs3849374 | 2 | 101443397 | *NPAS2, AC092168.2* | G | C | 0.821971 | 0.0161021 | 0.00261564 | 7.46E-10 | - | - | - | No | Yes |
| 51 | rs7569755 | 2 | 118648261 | *HTR5BP* | A | G | 0.29058 | 0.01425 | 0.00221206 | 1.18E-10 | - | - | - | No | Yes |
| 52 | rs13060130 | 3 | 84440527 | *AC108696.1* | C | T | 0.860311 | 0.0149893 | 0.00287224 | 1.80E-07 | - | - | - | No | Yes |
| 53 | rs9861009 | 3 | 141654685 | *TFDP2 (RP11-271K21.11)* | C | T | 0.727515 | 0.0140213 | 0.00225294 | 4.86E-10 | - | - | - | No | Yes |
| 54 | rs78649910 | 4 | 3482213 | *DOK7* | T | A | 0.893821 | 0.0211949 | 0.00325203 | 7.15E-11 | - | - | - | No | Yes |
| 55 | rs4364259 | 4 | 15892159 | *RP11-442P12.1* | A | G | 0.202148 | 0.0159119 | 0.00250595 | 2.16E-10 | - | - | - | No | Yes |
| 56 | rs4616820 | 4 | 57745481 | *REST* | C | T | 0.535046 | 0.012286 | 0.00201755 | 1.13E-09 | - | - | - | No | Yes |
| 57 | rs35057908 | 4 | 69372082 | *UGT2B29P* | T | A | 0.431309 | 0.0110085 | 0.00202343 | 5.31E-08 | - | - | - | No | Yes |
| 58 | rs13104260 | 4 | 70348090 | *UGT2B4* | A | G | 0.256938 | 0.0072531 | 0.00228347 | 0.00149139 | - | - | - | No | Yes |
| 59 | rs11732896 | 4 | 88287993 | *HSD17B11* | G | A | 0.701209 | 0.0160047 | 0.00217341 | 1.79E-13 | - | - | - | No | Yes |
| 60 | rs28364331 | 4 | 100201295 | *RP11-696N14.1, ADH1A* | G | A | 0.018086 | 0.068614 | 0.00747169 | 4.19E-20 | - | - | - | No | Yes |
| 61 | rs1229984 | 4 | 100239319 | *ADH1B* | T | C | 0.024889 | 0.0450574 | 0.00637113 | 1.53E-12 | - | - | - | No | Yes |
| 62 | rs10070734 | 5 | 87940026 | *LINC00461* | C | T | 0.709531 | 0.0132137 | 0.00219753 | 1.82E-09 | - | - | - | No | Yes |
| 63 | rs31612 | 5 | 108996643 | *AC012603.1* | T | C | 0.825562 | 0.014528 | 0.00264902 | 4.15E-08 | - | - | - | No | Yes |
| 64 | rs72834856 | 6 | 22801858 | *RP1-209A6.1* | T | G | 0.927936 | 0.0249871 | 0.00385103 | 8.67E-11 | - | - | - | No | Yes |
| 65 | rs28374650 | 6 | 32623367 | *HLA-DQB1* | C | T | 0.756438 | 0.0135623 | 0.00232502 | 5.44E-09 | - | - | - | No | Yes |
| 66 | rs9476310 | 6 | 57767576 | *RP11-325M4.2* | T | C | 0.511363 | 0.0117571 | 0.00200093 | 4.21E-09 | - | - | - | No | Yes |
| 67 | rs9490317 | 6 | 121859499 | *RNU4-76P* | C | T | 0.445894 | 0.011051 | 0.00201182 | 3.95E-08 | - | - | - | No | Yes |
| 68 | rs2248551 | 6 | 131924689 | *MED23* | G | A | 0.834778 | 0.0233623 | 0.0026823 | 3.04E-18 | - | - | - | No | Yes |
| 69 | rs10085881 | 7 | 21577960 | *DNAH11* | T | C | 0.717815 | 0.0145575 | 0.00223829 | 7.83E-11 | - | - | - | No | Yes |
| 70 | rs7784802 | 7 | 64015379 | *ZNF680* | T | A | 0.360991 | 0.0138131 | 0.00207209 | 2.62E-11 | - | - | - | No | Yes |
| 71 | rs6966728 | 7 | 104618318 | *LINC01004* | C | T | 0.537368 | 0.0117532 | 0.00203758 | 8.01E-09 | - | - | - | No | Yes |
| 72 | rs2346264 | 7 | 133536351 | *EXOC4* | A | C | 0.217315 | 0.0138826 | 0.00243596 | 1.21E-08 | - | - | - | No | Yes |
| 73 | rs34290760 | 8 | 9185179 | *RP11-115J16.1* | C | G | 0.970888 | 0.0334615 | 0.00593241 | 1.70E-08 | - | - | - | No | Yes |
| 74 | rs804281 | 8 | 11611865 | *GATA4* | G | A | 0.583605 | 0.0132996 | 0.00202139 | 4.72E-11 | - | - | - | No | Yes |
| 75 | rs28692966 | 8 | 25892919 | *EBF2* | A | G | 0.252936 | 0.0148311 | 0.00230018 | 1.14E-10 | - | - | - | No | Yes |
| 76 | rs2725371 | 8 | 30854033 | *PURG* | G | A | 0.697691 | 0.0118392 | 0.00218287 | 5.84E-08 | - | - | - | No | Yes |
| 77 | rs4738684 | 8 | 59393273 | *CYP7A1* | G | A | 0.665522 | 0.0124102 | 0.00211491 | 4.41E-09 | - | - | - | No | Yes |
| 78 | rs13284054 | 9 | 107669073 | *ABCA1* | C | T | 0.117727 | 0.0175688 | 0.00313411 | 2.07E-08 | - | - | - | No | Yes |
| 79 | rs10887718 | 10 | 82042624 | *MAT1A* | C | T | 0.471787 | 0.0111219 | 0.00199828 | 2.61E-08 | - | - | - | No | Yes |
| 80 | rs3925446 | 10 | 91495322 | *KIF20B* | A | G | 0.199129 | 0.0152166 | 0.00249607 | 1.09E-09 | - | - | - | No | Yes |
| 81 | rs4418728 | 10 | 94839724 | *CYP26A1* | T | G | 0.451688 | 0.0109611 | 0.00200006 | 4.24E-08 | - | - | - | No | Yes |
| 82 | rs61883501 | 11 | 13882754 | *RP11-98J9.2, RP11-98J9.3* | A | C | 0.966768 | 0.0017709 | 0.00553993 | 0.749232 | - | - | - | No | Yes |
| 83 | rs116970203 | 11 | 14876718 | *PDE3B* | G | A | 0.972844 | 0.376873 | 0.00612043 | 0 | - | - | - | No | Yes |
| 84 | rs117576073 | 11 | 14912573 | *CYP2R1* | G | T | 0.987265 | 0.147177 | 0.00886081 | 5.91E-62 | - | - | - | No | Yes |
| 85 | rs78168201 | 11 | 70971149 | *SHANK2* | T | C | 0.013766 | 0.0893469 | 0.00864963 | 5.18E-25 | - | - | - | No | Yes |
| 86 | rs964184 | 11 | 116648917 | *ZPR1 (ZNF259)* | C | G | 0.868376 | 0.0431755 | 0.00294423 | 1.09E-48 | - | - | - | No | Yes |
| 87 | rs613808 | 11 | 116710968 | *APOA1-AS* | G | A | 0.719993 | 0.0264476 | 0.00223913 | 3.40E-32 | - | - | - | No | Yes |
| 88 | rs2847500 | 11 | 120114421 | *POU2F3* | G | A | 0.876497 | 0.021925 | 0.00302751 | 4.42E-13 | - | - | - | No | Yes |
| 89 | rs12317268 | 12 | 21352541 | *SLCO1B1* | A | G | 0.848996 | 0.0208967 | 0.00278474 | 6.19E-14 | - | - | - | No | Yes |
| 90 | rs11182428 | 12 | 38526387 | *RNA5SP358* | T | C | 0.480005 | 0.0125352 | 0.00199379 | 3.23E-10 | - | - | - | No | Yes |
| 91 | rs1038165 | 12 | 68665940 | *MDM1* | T | C | 0.583349 | 0.0120567 | 0.002018 | 2.31E-09 | - | - | - | No | Yes |
| 92 | rs11108368 | 12 | 96386138 | *HAL* | G | A | 0.606145 | 0.0038307 | 0.00209129 | 0.0669916 | - | - | - | No | Yes |
| 93 | rs12372115 | 12 | 97982701 | *RMST* | G | T | 0.929281 | 0.0217954 | 0.00387948 | 1.93E-08 | - | - | - | No | Yes |
| 94 | rs73413596 | 12 | 111582630 | *CUX2* | C | T | 0.073854 | 0.0216996 | 0.00382584 | 1.41E-08 | - | - | - | No | Yes |
| 95 | rs7149014 | 14 | 29802911 | *RP11-562L8.1* | T | C | 0.370807 | 0.0129475 | 0.00208615 | 5.42E-10 | - | - | - | No | Yes |
| 96 | rs12881545 | 14 | 101176212 | *DLK1* | C | G | 0.673439 | 0.011822 | 0.00213142 | 2.91E-08 | - | - | - | No | Yes |
| 97 | rs1800588 | 15 | 58723675 | *ALDH1A2, LIPC* | C | T | 0.784797 | 0.0329215 | 0.00242187 | 4.38E-42 | - | - | - | No | Yes |
| 98 | rs55829990 | 15 | 63790642 | *USP3* | T | C | 0.655996 | 0.0186013 | 0.00210294 | 9.12E-19 | - | - | - | No | Yes |
| 99 | rs62007299 | 15 | 77711719 | *PEAK1* | G | A | 0.287463 | 0.0133407 | 0.00219977 | 1.32E-09 | - | - | - | No | Yes |
| 100 | rs325384 | 15 | 100229761 | *MEF2A* | C | T | 0.715795 | 0.0141728 | 0.00221789 | 1.66E-10 | - | - | - | No | Yes |
| 101 | rs17231506 | 16 | 56994528 | *CETP* | C | T | 0.676894 | 0.0184236 | 0.00213148 | 5.45E-18 | - | - | - | No | Yes |
| 102 | rs11076175 | 16 | 57006378 | *CETP* | G | A | 0.178358 | 0.0230493 | 0.00260705 | 9.47E-19 | - | - | - | No | Yes |
| 103 | rs4327060 | 16 | 72807438 | *RP5-991G20.1* | C | T | 0.945604 | 0.0243589 | 0.00439221 | 2.92E-08 | - | - | - | No | Yes |
| 104 | rs4575545 | 16 | 79755446 | *RP11-345M22.1, RP11-345M22.2* | G | A | 0.695172 | 0.0155823 | 0.00217312 | 7.47E-13 | - | - | - | No | Yes |
| 105 | rs11542462 | 16 | 82033810 | *SDR42E1* | G | A | 0.865656 | 0.023334 | 0.00291776 | 1.27E-15 | - | - | - | No | Yes |
| 106 | rs10454087 | 17 | 40735641 | *RETREG3 (FAM134C)* | C | T | 0.715178 | 0.0135306 | 0.00220667 | 8.70E-10 | - | - | - | No | Yes |
| 107 | rs2952289 | 17 | 66464414 | *RP11-120M18.2* | T | C | 0.798032 | 0.017715 | 0.00249217 | 1.18E-12 | - | - | - | No | Yes |
| 108 | rs8091117 | 18 | 28919794 | *DSG1* | C | A | 0.934702 | 0.0263626 | 0.0040284 | 5.98E-11 | - | - | - | No | Yes |
| 109 | rs4121823 | 18 | 47144223 | *LIPG* | T | A | 0.154667 | 0.0192879 | 0.00277797 | 3.83E-12 | - | - | - | No | Yes |
| 110 | rs590215 | 18 | 57904088 | *RP11-795H16.2* | C | T | 0.734073 | 0.0129217 | 0.00225753 | 1.04E-08 | - | - | - | No | Yes |
| 111 | rs2037511 | 18 | 61366207 | *SERPINB11* | A | G | 0.166007 | 0.0181228 | 0.00267963 | 1.35E-11 | - | - | - | No | Yes |
| 112 | rs142158911 | 19 | 11190534 | *LDLR* | A | G | 0.114608 | 0.0255317 | 0.00314553 | 4.79E-16 | - | - | - | No | Yes |
| 113 | rs187429064 | 19 | 19380513 | *AC138430.4, TM6SF2* | G | A | 0.011266 | 0.0648324 | 0.00947915 | 7.95E-12 | - | - | - | No | Yes |
| 114 | rs3814995 | 19 | 36342212 | *NPHS1* | C | T | 0.688405 | 0.012558 | 0.00214992 | 5.18E-09 | - | - | - | No | Yes |
| 115 | rs7412 | 19 | 45412079 | *APOE* | T | C | 0.082073 | 0.0300485 | 0.00363434 | 1.36E-16 | - | - | - | No | Yes |
| 116 | rs484195 | 19 | 45421877 | *APOC1* | A | G | 0.384386 | 0.0155156 | 0.00209699 | 1.37E-13 | - | - | - | No | Yes |
| 117 | rs8113404 | 19 | 53065579 | *ZNF808, ZNF701* | T | C | 0.304586 | 0.012173 | 0.00217136 | 2.07E-08 | - | - | - | No | Yes |
| 118 | rs11606 | 19 | 54658102 | *CNOT3* | G | C | 0.425162 | 0.0120363 | 0.00205119 | 4.41E-09 | - | - | - | No | Yes |
| 119 | rs2207132 | 20 | 39142516 | *MAFB* | G | A | 0.96711 | 0.0345955 | 0.00557773 | 5.56E-10 | - | - | - | No | Yes |
| 120 | rs2229742 | 21 | 16339172 | *NRIP1* | G | C | 0.896549 | 0.0251483 | 0.00327069 | 1.48E-14 | - | - | - | No | Yes |
| 121 | rs6003456 | 22 | 23356100 | *AP000362.1* | T | A | 0.765336 | 0.013277 | 0.00236378 | 1.95E-08 | - | - | - | No | Yes |
| 122 | rs115621755 | 22 | 50853134 | *PPP6R2* | C | T | 0.67288 | 0.0124309 | 0.00212296 | 4.76E-09 | - | - | - | No | Yes |

A1: serum-25(OH)D-increasing allele; A2: alternative allele; A1F: allele frequency for A1; SE: standard error; 25(OH)D: 25-hydrovimtain D; SNP: single nucleotide polymorphism; CHR: chromosome number; BP: base-pair position, Genome Reference Consortium Human Build 37 (GRCh37); vitaminD-GS: genetic instrument (using 35 GWAS variants) for serum 25(OH)D concentration used in the primary analysis; vitaminD-GS-122: genetic instrument (using 122 GWAS variants) for serum 25(OH)D concentration used in the sensitivity analysis; ^a^obtained from Revez, 2020 et al ^3^.; **^b^**imputed summary statistics, obtained from Revez, 2020 et al ^3^. Serum 25(OH)D has been natural-log transformed; ^c^SNP proxy in the SUNLIGHT consortium: rs1123571, r^2^ = 0.86806 (1000 Genome, EUR); ^d^SNP proxy in the SUNLIGHT consortium: rs2186777, r^2^ = 1 (1000 Genome, EUR).

**Table S2. Genomewide significant CRP variants used for the genetic instruments for serum CRP concentrations.**

| **No** | **SNP** | **CHR** | **BP** | **Gene** | **A1** | **A2** | **A1F** | **Beta^a^** | **SE** | ***P*** | **SNPs for**  **CRP-gwasGS^b^** | **SNPs for**  **CRP-cisGS^c^** |
| --- | --- | --- | --- | --- | --- | --- | --- | --- | --- | --- | --- | --- |
| 1 | rs2794520 | 1 | 159678816 | *CRP* | C | T | 0.33 | 0.182 | 0.004 | 4.17E-523 | Yes | No |
| 2 | rs4129267 | 1 | 154426264 | *IL6R* | C | T | 0.39 | 0.088 | 0.004 | 1.20E-129 | Yes | No |
| 3 | rs1805096 | 1 | 66102257 | *LEPR* | G | A | 0.39 | 0.104 | 0.004 | 2.17E-183 | Yes | No |
| 4 | rs10925027 | 1 | 247612562 | *NLRP3* | T | C | 0.4 | 0.036 | 0.004 | 4.25E-21 | Yes | No |
| 5 | rs2293476 | 1 | 40036847 | *PABPC4* | C | G | 0.23 | 0.03 | 0.004 | 8.27E-13 | Yes | No |
| 6 | rs469772 | 1 | 91530305 | *ZNF644* | C | T | 0.81 | 0.031 | 0.005 | 5.54E-12 | Yes | No |
| 7 | rs4246598 | 2 | 88438050 | *FABP1* | A | C | 0.46 | 0.022 | 0.004 | 5.11E-10 | Yes | No |
| 8 | rs1260326 | 2 | 27730940 | *GCKR* | T | C | 0.39 | 0.073 | 0.004 | 2.72E-92 | Yes | No |
| 9 | rs1441169 | 2 | 214033530 | *IKZF2* | A | G | 0.47 | 0.025 | 0.004 | 2.27E-11 | Yes | No |
| 10 | rs13409371 | 2 | 113838145 | *IL1F10* | A | G | 0.43 | 0.048 | 0.004 | 5.07E-36 | Yes | No |
| 11 | rs9284725 | 2 | 102744854 | *IL1R1* | C | A | 0.24 | 0.027 | 0.004 | 7.34E-11 | Yes | No |
| 12 | rs12995480 | 2 | 629881 | *TMEM18* | C | T | 0.83 | 0.031 | 0.005 | 1.24E-10 | Yes | No |
| 13 | rs2352975 | 3 | 49891885 | *TRAIP* | C | T | 0.3 | 0.025 | 0.004 | 6.43E-10 | Yes | No |
| 14 | rs17658229 | 5 | 172191052 | *DUSP1* | C | T | 0.05 | 0.056 | 0.01 | 5.50E-09 | Yes | No |
| 15 | rs1490384 | 6 | 126851160 | *C6orf173* | C | T | 0.49 | 0.025 | 0.004 | 2.65E-12 | Yes | No |
| 16 | rs12202641 | 6 | 116314634 | *FRK* | C | T | 0.61 | 0.023 | 0.004 | 3.00E-10 | Yes | No |
| 17 | rs9271608 | 6 | 32591588 | *HLA-DQA1* | G | A | 0.22 | 0.042 | 0.005 | 2.33E-17 | Yes | No |
| 18 | rs9385532 | 6 | 130371227 | *L3MBTL3* | C | T | 0.67 | 0.026 | 0.004 | 1.90E-11 | Yes | No |
| 19 | rs13233571 | 7 | 72971231 | *BCL7B* | C | T | 0.12 | 0.057 | 0.005 | 2.95E-25 | Yes | No |
| 20 | rs1880241 | 7 | 22759469 | *IL6* | A | G | 0.52 | 0.028 | 0.004 | 8.41E-14 | Yes | No |
| 21 | rs2710804 | 7 | 36084529 | *KIAA1706* | C | T | 0.37 | 0.021 | 0.004 | 1.30E-08 | Yes | No |
| 22 | rs2891677 | 8 | 126344208 | *NSMCE2* | T | C | 0.54 | 0.02 | 0.004 | 1.59E-08 | Yes | No |
| 23 | rs4841132 | 8 | 9183596 | *PPP1R3B* | G | A | 0.09 | 0.065 | 0.006 | 2.00E-25 | Yes | No |
| 24 | rs2064009 | 8 | 117007850 | *TRPS1* | T | C | 0.58 | 0.027 | 0.004 | 2.28E-14 | Yes | No |
| 25 | rs643434 | 9 | 136142355 | *ABO* | A | G | 0.37 | 0.023 | 0.004 | 1.02E-09 | Yes | No |
| 26 | rs1051338 | 10 | 91007360 | *LIPA* | G | T | 0.31 | 0.024 | 0.004 | 2.27E-09 | Yes | No |
| 27 | rs10832027 | 11 | 13357183 | *ARNTL* | A | G | 0.67 | 0.026 | 0.004 | 4.43E-12 | Yes | No |
| 28 | rs10838687 | 11 | 47312892 | *MADD* | T | G | 0.78 | 0.031 | 0.004 | 9.12E-13 | Yes | No |
| 29 | rs1582763 | 11 | 60021948 | *MS4A4A* | G | A | 0.63 | 0.022 | 0.004 | 2.37E-09 | Yes | No |
| 30 | rs10778215 | 12 | 103537266 | *ASCL1* | T | A | 0.49 | 0.033 | 0.004 | 1.86E-20 | Yes | No |
| 31 | rs7310409 | 12 | 121424861 | *HNF1A* | G | A | 0.39 | 0.137 | 0.004 | 2.54E-299 | Yes | No |
| 32 | rs2239222 | 14 | 73011885 | *RGS6* | G | A | 0.36 | 0.035 | 0.004 | 9.87E-20 | Yes | No |
| 33 | rs4774590 | 15 | 51745277 | *DMXL2* | G | A | 0.65 | 0.022 | 0.004 | 2.71E-08 | Yes | No |
| 34 | rs340005 | 15 | 60878030 | *RORA* | A | G | 0.38 | 0.03 | 0.004 | 1.01E-15 | Yes | No |
| 35 | rs1558902 | 16 | 53803574 | *FTO* | A | T | 0.41 | 0.034 | 0.004 | 5.20E-20 | Yes | No |
| 36 | rs10521222 | 16 | 51158710 | *SALL1* | C | T | 0.05 | 0.104 | 0.011 | 2.06E-22 | Yes | No |
| 37 | rs10512597 | 17 | 72699833 | *CD300LF, RAB37* | C | T | 0.82 | 0.037 | 0.005 | 4.44E-14 | Yes | No |
| 38 | rs178810 | 17 | 16097430 | *NCOR1* | T | C | 0.56 | 0.02 | 0.004 | 2.95E-08 | Yes | No |
| 39 | rs12960928 | 18 | 57897803 | *MC4R* | C | T | 0.27 | 0.024 | 0.004 | 1.91E-09 | Yes | No |
| 40 | rs4092465 | 18 | 55080437 | *ONECUT2* | G | A | 0.65 | 0.027 | 0.004 | 3.11E-10 | Yes | No |
| 41 | rs2852151 | 18 | 12841176 | *PTPN2* | A | G | 0.4 | 0.025 | 0.004 | 1.36E-11 | Yes | No |
| 42 | rs4420638 | 19 | 45422946 | *APOC1* | A | G | 0.18 | 0.229 | 0.006 | 1.23E-305 | Yes | No |
| 43 | rs1800961 | 20 | 43042364 | *HNF4A* | C | T | 0.03 | 0.112 | 0.011 | 4.63E-23 | Yes | No |
| 44 | rs2315008 | 20 | 62343956 | *ZGPAT* | G | T | 0.69 | 0.023 | 0.004 | 5.36E-10 | Yes | No |
| 45 | rs2836878 | 21 | 40465534 | *DSCR2* | G | A | 0.27 | 0.043 | 0.004 | 7.71E-26 | Yes | No |
| 46 | rs6001193 | 22 | 39074737 | *TOMM22* | A | G | 0.65 | 0.028 | 0.004 | 6.53E-14 | Yes | No |
| 47 | rs3093077^c^ | 1 | 159679636 | *CRP* | C | A | 0.17 | - | - | - | No | Yes |
| 48 | rs1205^c^ | 1 | 159682233 | *CRP* | C | T | 0.70 | - | - | - | No | Yes |
| 49 | rs1130864^c^ | 1 | 159683091 | *CRP* | A | G | 0.26 | - | - | - | No | Yes |
| 50 | rs1800947^c^ | 1 | 159683438 | *CRP* | C | G | 0.96 | - | - | - | No | Yes |

A1: serum-CRP-increasing allele; A2: alternative allele; A1F: allele frequency for A1; SE: standard error; SNP: single nucleotide polymorphism; CHR: chromosome number; BP: base-pair position, Genome Reference Consortium Human Build 37 (GRCh37); CRP” C-reactive protein; CRP-gwasGS: genetic instrument for serum CRP using 46 GWAS variants; CRP-cisGS: genetic instrument for serum CRP using cis variants; ^a^change in natural-log-transformed CRP (mg/L) per copy increment in alele A1; ^b^identified from Ligthart, 2018 et al. ^14^; ^c^identified from Robinson,T 2020 et al ^15^.

**Table S3.** **Association of vitaminD-GS and CRP-gwasGS with potential confounders in the UK Biobank.**

|  |  | **VitaminD-GS** | **CRP-gwasGS** |
| --- | --- | --- | --- |
|  | N (%) | Mean (SD) | Mean (SD) |
| **BMI** |  |  |  |
| <18.5 kg/m^2^ | 1,443 (0.49) | 38.40 (5.91) | 57.05 (5.37) |
| [18.5, 25) kg/m^2^ | 96,053 (32.66) | 38.42 (5.88) | 57.05 (5.43) |
| [25, 30) kg/m^2^ | 125,559 (42.69) | 38.42 (5.88) | 57.17 (5.42) |
| ≥30 kg/m^2^ | 71,037 (24.15) | 38.46 (5.86) | 57.26 (5.41) |
| *P*^a^ |  | 0.85 | 1.066E-13 |
| **Smoking** |  |  |  |
| Non-smokers | 160,897 (54.74) | 38.42 (5.86) | 57.14 (5.41) |
| Ex-smokers | 103,600 (35.24) | 38.45 (5.89) | 57.15 (5.45) |
| Smokers^b^ | 7,561 (2.57) | 38.50 (5.88) | 57.16 (5.37) |
| Cigars/Pipes | 1,682 (0.57) | 38.39 (5.97) | 57.14 (5.37) |
| <1 to 15 cigs/day | 12,056 (4.1) | 38.33 (5.89) | 57.25 (5.46) |
| >15 cigs/day | 8,158 (2.78) | 38.39 (5.86) | 57.22 (5.32) |
| *P*^a^ |  | 0.10 | 0.23 |
| **Alcohol intake** |  |  |  |
| Non-drinkers | 19,178 (6.51) | 38.51 (5.86) | 57.16 (5.39) |
| Special occasions or 1-3 times/month | 63,647 (21.59) | 38.38 (5.88) | 57.20 (5.41) |
| 1 or 2 times/week | 78,199 (26.53) | 38.46 (5.87) | 57.15 (5.42) |
| 3 or 4 times/week | 71,296 (24.19) | 38.46 (5.87) | 57.14 (5.43) |
| Daily or almost daily | 62,445 (21.18) | 38.39 (5.88) | 57.12 (5.42) |
| *P*^a^ |  | 0.051 | 0.082 |
| **Physical activity** |  |  |  |
| Light | 88,245 (30.60) | 38.45 (5.87) | 57.20 (5.41) |
| Moderate | 142,803 (49.51) | 38.42 (5.88) | 57.15 (5.42) |
| Vigorous | 57,373 (19.89) | 38.42 (5.87) | 57.10 (5.42) |
| *P*^a^ |  | 0.55 | 0.004 |
| **Education** |  |  |  |
| None | 50,706 (17.34) | 38.45 (5.86) | 57.14 (5.4) |
| NVQ/CSE/A-levels | 105,433 (36.05) | 38.43 (5.88) | 57.15 (5.42) |
| Degree/professional | 136,357 (46.62) | 38.42 (5.87) | 57.16 (5.43) |
| *P*^a^ |  | 0.50 | 0.80 |
| **Townsend deprivation index quartiles** |  |  |  |
| Q1 lowest | 73,533 (24.96) | 38.41 (5.85) | 57.14 (5.42) |
| Q2 | 73,780 (25.04) | 38.46 (5.89) | 57.15 (5.43) |
| Q3 | 73,655 (25.0) | 38.42 (5.87) | 57.16 (5.42) |
| Q4 highest | 73,658 (25.0) | 38.44 (5.88) | 57.15 (5.41) |
| *P*^a^ |  | 0.40 | 0.95 |

NVQ, National Vocational Qualification; CSE, Certificate of Secondary Education; A-levels, Advanced levels; SD, standard deviation; Q, quartiles; cig, cigarette.

^a^*P* values have been adjusted for age, sex, genotyping array, birth location, and assessment center.

^b^current smokers without information on types of tobacco that they smoke.

**Table S4.** **Genetic association of serum CRP with 25(OH)D concentrations**

|  |  | **One-sample linear MR** | | **Non-linear MR** |
| --- | --- | --- | --- | --- |
|  | N | Beta (95% CI)^a^ | *P_linear_* | *P_non-linear_*^b^ |
| CRP-gwasGS | 294,970 | -0.14 (-0.40, 0.13) | 0.32 | 0.76^c^ |
| CRP-cisGS | 291,738 | -0.21 (-0.70, 0.28) | 0.40 | 0.40 |
| CRP-gwasGS (BMI adjustment) | 294,092 | 0.079 (-0.19, 0.35) | 0.57 | 0.94 |
| CRP-gwasGS (Physical activity adjustment) | 288,421 | -0.059 (-0.33, 0.21) | 0.67 | 0.57 |

^a^per 1 unit increase in log CRP concentration (log(mg/L)).

^b^Likelihood ratio test comparing the besting-fitting fractional polynomial model against the linear model

^c^*P_non-linear_* = 0.79, after excluding local causal estimates from the first and 100^th^ stratum of residuals of serum logCRP (Supplementary Figure S5).

CRP: C-reactive protein; 25(OH)D: 25-hydroxyvitamin D; BMI: body mass index.

**Table S5. Genetic variants associated with metabolic traits**

| **SNP^a^** | **Gene** | **Metabolic Traits** |
| --- | --- | --- |
| rs76798800 | *DCST2* | Various, incl. fat-free mass, birth weight, height |
| rs78151190 | *CARMIL1 (LRRC16A)* | HbA1c, pulse, blood pressure |
| rs77924615 | *PDILT* | Blood pressure |
| rs261291 | *ALDH1A2* | Cholesterol, lipid metabolism |
| rs727857 | *LINC01122* | Fat mass, BMI, impedance, weight |
| rs1047891 | *CPS1* | Various, incl. weight, impedance, fat- free mass cholesterol |
| rs12794714 | *CYP2R1* | Hip circumference |
| rs72997623 | *DGAT2* | HDL cholesterol |
| rs7528419 | *CELSR2* | Angina pectoris, coronary artery disease, cholesterol, statin use |
| rs75741381 | *VGF* | Impedance |
| rs6782190 | *CADM2* | Fat free mass, BMI, impedance |

^a^identified using trait associations identified through PhenoScanner V2 ^5^.

**Table S6. Genetic variants used to construct the focused score**

| **No** | **SNP** | **CHR** | **BP** | **Gene** | **A1** | **A2** | **Beta^a^** |
| --- | --- | --- | --- | --- | --- | --- | --- |
| 1 | rs1352846 | 4 | 72617775 | *GC* | A | G | 0.172 |
| 2 | rs7041 | 4 | 72618334 | *GC* | C | A | 0.045 |
| 3 | rs4694431 | 4 | 72634343 | *GC* | T | C | 0.034 |
| 4 | rs139148694 | 4 | 72770563 | *GC* | G | GTGCTTTTATCAA | 0.028 |
| 5 | rs16913816 | 11 | 14339328 | *CYP2R1* | A | G | 0.031 |
| 6 | rs117913124 | 11 | 14900931 | *CYP2R1* | G | A | 0.503 |
| 7 | rs117576073 | 11 | 14912573 | *CYP2R1* | G | T | 0.246 |
| 8 | rs12794714 | 11 | 14913575 | *CYP2R1* | G | A | 0.139 |
| 9 | rs202122669 | 11 | 14913645 | *CYP2R1* | A | G | 0.615 |
| 10 | rs187639972 | 11 | 14913900 | *CYP2R1* | C | G | 0.36 |
| 11 | rs117115472 | 11 | 14941652 | *CYP2R1* | C | G | 0.148 |
| 12 | rs139168803 | 11 | 71157867 | *DHCR7* | A | G | 0.188 |
| 13 | rs12573951 | 11 | 71158672 | *DHCR7* | G | A | 0.045 |
| 14 | rs7928249 | 11 | 71161063 | *DHCR7* | G | A | 0.131 |
| 15 | rs549000212 | 11 | 71180762 | *DHCR7* | A | C | 0.364 |
| 16 | rs4081429 | 11 | 71290740 | *DHCR7* | A | C | 0.017 |
| 17 | rs6123359 | 20 | 52714706 | *CYP24A1* | G | A | 0.026 |
| 18 | rs6127099 | 20 | 52731402 | *CYP24A1* | A | T | 0.013 |
| 19 | rs35870583 | 20 | 52735238 | *CYP24A1* | G | GT | 0.027 |
| 20 | rs2585442 | 20 | 52737123 | *CYP24A1* | G | C | 0.025 |
| 21 | rs2762942 | 20 | 52788925 | *CYP24A1* | A | G | 0.053 |

A1: serum-25(OH)D-increasing allele; A2: alternative allele; SNP: single nucleotide polymorphism; CHR: chromosome number; BP: base-pair position, Genome Reference Consortium Human Build 37 (GRCh37).

^a^Conditional association with 25(OH)D (nmol/L), obtained from Sofianopoulou,E, 2021 et al ^6^.

**References**

1. Fry D, Almond R, Moffat S, Gordon M, Singh P. Companion Document to Accompany Serum Biomarker Data. Version 1.0 (11/03/2019). https://biobank.ndph.ox.ac.uk/showcase/showcase/docs/serum_biochemistry.pdf (accessed 30/11/2021).

2. UK Biobank. Biomarker assay quality procedures: approaches used to minimise systematic and random errors (and the wider epidemiological implications). Version 1.2 (02/04/2019). https://biobank.ndph.ox.ac.uk/showcase/ukb/docs/biomarker_issues.pdf (accessed 30/11/2021).

3. Revez JA, Lin T, Qiao Z, et al. Genome-wide association study identifies 143 loci associated with 25 hydroxyvitamin D concentration. *Nat Commun*. 2020 Apr 2;**11**(1):1647.

4. Burgess S, Thompson SG. Use of allele scores as instrumental variables for Mendelian randomization. *Int J Epidemiol*. 2013/09/26 ed. 2013 Aug;**42**(4):1134–44.

5. Kamat MA, Blackshaw JA, Young R, et al. PhenoScanner V2: an expanded tool for searching human genotype-phenotype associations. *Bioinformatics*. 2019/06/25 ed. 2019 Jun 24;

6. Sofianopoulou E, Kaptoge SK, Afzal S, et al. Estimating dose-response relationships for vitamin D with coronary heart disease, stroke, and all-cause mortality: observational and Mendelian randomisation analyses. *Lancet Diabetes Endocrinol*. 2021 Dec 1;**9**(12):837–846.

7. Vimaleswaran KS, Cavadino A, Berry DJ, et al. Association of vitamin D status with arterial blood pressure and hypertension risk: a mendelian randomisation study. *Lancet Diabetes Endocrinol*. 2014 Sep 1;**2**(9):719–729.

8. Vimaleswaran KS, Berry DJ, Lu C, et al. Causal relationship between obesity and vitamin D status: bi-directional Mendelian randomization analysis of multiple cohorts. *PLoS Med*. 2013;**10**(2):e1001383.

9. Bowden J, Del Greco MF, Minelli C, Davey Smith G, Sheehan N, Thompson J. A framework for the investigation of pleiotropy in two-sample summary data Mendelian randomization. *Stat Med*. 2017/01/24 ed. 2017 May 20;**36**(11):1783–1802.

10. Bowden J, Davey Smith G, Burgess S. Mendelian randomization with invalid instruments: effect estimation and bias detection through Egger regression. *Int J Epidemiol*. 2015/06/08 ed. 2015 Apr;**44**(2):512–25.

11. Bowden J, Davey Smith G, Haycock PC, Burgess S. Consistent Estimation in Mendelian Randomization with Some Invalid Instruments Using a Weighted Median Estimator. *Genet Epidemiol*. 2016/04/12 ed. 2016 May;**40**(4):304–14.

12. Hartwig FP, Davey Smith G, Bowden J. Robust inference in summary data Mendelian randomization via the zero modal pleiotropy assumption. *Int J Epidemiol*. 2017/10/19 ed. 2017 Dec 1;**46**(6):1985–1998.

13. Verbanck M, Chen C-Y, Neale B, Do R. Detection of widespread horizontal pleiotropy in causal relationships inferred from Mendelian randomization between complex traits and diseases. *Nat Genet*. 2018 May;**50**(5):693–698.

14. Ligthart S, Vaez A, Võsa U, et al. Genome Analyses of >200,000 Individuals Identify 58 Loci for Chronic Inflammation and Highlight Pathways that Link Inflammation and Complex Disorders. *Am J Hum Genet*. 2018 Nov 1;**103**(5):691–706.

15. Robinson T, Martin RM, Yarmolinsky J. Mendelian randomisation analysis of circulating adipokines and C-reactive protein on breast cancer risk. *Int J Cancer*. 2020;**147**(6):1597–1603.
